# Circulating tumor DNA in neoadjuvant therapy for solid tumors

**DOI:** 10.3389/fonc.2026.1909934

**Published:** 2026-08-26

**Authors:** Xueqin Huang, Yu Deng, Yi Shen

**Affiliations:** 1 Hemodialysis Center, Department of Nephrology, West China Hospital, Sichuan University, Chengdu, China; 2 Department of Thoracic Surgery, West China Hospital, Sichuan University, Chengdu, China; 3 Department of Thoracic Surgery, West China School of Public Health and West China Fourth Hospital, Sichuan University, Chengdu, China

**Keywords:** circulating tumor DNA, efficacy prediction, liquid biopsy, minimal residual disease, neoadjuvant therapy, recurrence monitoring, solid tumors

## Abstract

Circulating tumor DNA (ctDNA), a core component of liquid biopsy, demonstrates significant potential in the field of neoadjuvant therapy for solid tumors. This review systematically examines the role of ctDNA across the pre-, intra-, and post-neoadjuvant treatment phases, with a focus on its value in predicting therapeutic efficacy, assessing early treatment response, detecting minimal residual disease (MRD), and monitoring for recurrence. By synthesizing the latest clinical research data and advancements in molecular detection technologies, this article aims to elucidate how ctDNA is facilitating a shift towards more precise, dynamic, and individualized paradigms in neoadjuvant therapy for solid tumors. Furthermore, it analyzes the current challenges and future directions for integrating ctDNA analysis into clinical practice to optimize patient management and outcomes. Throughout, clinically validated applications are explicitly distinguished from those that remain investigational, and key unresolved questions are highlighted.

## 1 Introduction

Neoadjuvant therapy has become a cornerstone in the multimodal management of various solid tumors, including breast, colorectal, lung, and esophageal cancers, with the primary goals of downstaging disease, improving surgical resectability, and ultimately enhancing patient survival (1). However, conventional imaging (CT/MRI) and post-treatment pathological evaluation of the surgical specimen provide only static, retrospective snapshots of tumor response: they offer no real-time insight during the critical “black box” period of active therapy, in which patients may be exposed to ineffective yet toxic treatment; they cannot capture the molecular heterogeneity of tumors evolving under therapeutic pressure; and repeated tissue biopsies are invasive (1). This landscape underscores a pressing clinical need for a non-invasive, sensitive, and specific biomarker capable of providing a dynamic, molecularly informed assessment of tumor burden and treatment response throughout the neoadjuvant journey.

Circulating tumor DNA (ctDNA), comprising fragmented DNA shed by tumor cells into the bloodstream, has emerged as a tool well suited to address these limitations. Its short half-life of approximately two hours enables it to serve as a real-time, liquid biopsy reflecting the current genomic landscape and burden of the tumor (1). Advances in high-throughput sequencing and bioinformatic error suppression now allow tumor-specific genetic and epigenetic alterations to be detected at very low allele frequencies, and ctDNA analysis has consequently moved beyond genotyping of advanced disease toward monitoring of minimal residual disease (MRD) and prediction of treatment outcomes in the curative-intent, non-metastatic setting (2). The clinical promise of ctDNA lies in its ability to provide a serial, minimally invasive window into tumor dynamics, offering the potential to guide personalized therapeutic decisions much earlier than conventional methods allow.

The application of ctDNA in the neoadjuvant setting spans the entire treatment continuum, from baseline assessment through on-treatment monitoring to post-therapy surveillance for recurrence. At diagnosis, the presence and level of ctDNA can provide prognostic information and a tumor-specific molecular fingerprint. During active neoadjuvant therapy, the kinetics of ctDNA—specifically its clearance or persistence—have been strongly correlated with pathological response and long-term survival outcomes across multiple cancer types. For instance, rapid clearance of ctDNA is consistently associated with favorable pathological complete response (pCR) and improved survival, while persistent detectable ctDNA mid-treatment robustly identifies patients with a poor response who are at high risk of recurrence. This dynamic information creates a rationale for response-adapted treatment, and in the post-treatment phase ctDNA-based MRD detection identifies patients at elevated risk of recurrence, often months before radiographic relapse, providing a potential window for intervention. This review examines the evidence for ctDNA across the entire neoadjuvant continuum, from baseline assessment through on-treatment monitoring to postoperative MRD evaluation and recurrence surveillance. Rather than cataloguing individual studies, we aim to synthesize the evidence critically, to highlight where findings are robust, conflicting, or still preliminary, and to distinguish explicitly between clinically validated applications and those that remain investigational.

## 2 ctDNA detection technology platforms and their optimization in the context of neoadjuvant therapy

### 2.1 Mainstream detection technologies comparison and selection

The landscape of ctDNA detection technologies is broadly divided into tumor-informed and tumor-agnostic (or tumor-uninformed) approaches, each with distinct advantages, limitations, and clinical applicability (3). Tumor-informed assays require prior access to tumor tissue, typically through whole-exome or whole-genome sequencing, to construct a patient-specific mutation profile (4). This personalized panel is then used to track these specific mutations in serial plasma samples, offering high analytical sensitivity and specificity, particularly for monitoring known clonal mutations in MRD settings (5). For instance, the Invitae Personalized Cancer Monitoring™ assay, which utilizes whole exome sequencing of tumor tissue to design patient-specific panels, demonstrated high sensitivity (96.3%) and specificity (>99.9%) for detecting ctDNA at variant allele frequencies as low as 0.008% (4). Similarly, other tumor-informed platforms like RaDaR and NeXT Personal have shown the ability to detect ctDNA at parts-per-million levels, enabling ultrasensitive monitoring for recurrence prediction in early-stage cancers such as lung adenocarcinoma (6, 7). However, this approach is inherently complex, costly, dependent on the availability and quality of a tumor tissue sample, and involves longer turnaround times, which can limit its feasibility in rapid clinical decision-making scenarios (2, 3).

In contrast, tumor-agnostic assays directly analyze plasma cell-free DNA using fixed gene panels without prior knowledge of the tumor’s mutational landscape (3). These panels can range from small, focused sets targeting common oncogenic hotspots (e.g., in *EGFR*, *KRAS*, *BRAF*) to larger comprehensive genomic profiling panels covering hundreds of genes (8, 9). The primary advantage of this approach is its independence from tumor tissue, allowing for broader application, faster turnaround, and the potential to detect unknown or emerging mutations (2). Techniques such as droplet digital PCR (ddPCR) and BEAMing offer high sensitivity for detecting specific mutant alleles and are widely used for this purpose (9, 10). For example, ddPCR assays have been successfully applied for *EGFR* mutation detection in non-small cell lung cancer (NSCLC) and for monitoring MRD in colorectal cancer (11, 12). However, tumor-agnostic methods face significant challenges, including higher background noise from clonal hematopoiesis and non-malignant sources, which can complicate interpretation (8). Their sensitivity for detecting very low-frequency ctDNA, especially in early-stage disease or post-curative treatment settings where tumor shedding is minimal, is generally lower compared to tumor-informed assays (3, 13). This is evidenced by studies showing that tumor-informed assays often outperform tumor-agnostic ones in MRD detection sensitivity (14). The choice between these strategies depends on multiple factors, including the clinical question (e.g., therapy selection vs. MRD monitoring), disease stage, availability of tissue, cost-effectiveness, and the level of evidence from ongoing clinical trials (2, 3).

Beyond mutation-based profiling, the analysis of ctDNA-specific methylation patterns represents a rapidly evolving and promising technology (15). Methylation assays exploit the fact that cancer cells exhibit distinct, often tissue-specific, DNA methylation profiles compared to normal cells (16). These epigenetic markers offer several potential advantages: they provide strong tissue-of-origin signals, which can be crucial for cancers of unknown primary; they are abundant across the genome, allowing for the detection of multiple markers from a single DNA molecule; and they hold significant promise for early cancer detection due to their occurrence early in carcinogenesis (17, 18). Techniques for methylation analysis include bisulfite conversion followed by sequencing or PCR-based methods like methylation-specific ddPCR (MS-ddPCR) (19, 20). For instance, a multiplex MS-ddPCR assay targeting tumor-specific and tissue-conserved methylation markers demonstrated high specificity (96.7%) and sensitivity for detecting ctDNA in colorectal cancer patients, with ctDNA dynamics strongly correlating with progression-free and overall survival in metastatic disease (19). Furthermore, assays like MeD-Seq (genome-wide methylation profiling) and the tumor-naïve Northstar Response™ assay, which quantifies methylated ctDNA, are being explored for therapy response monitoring across various solid tumors (18, 21). The application of methylation analysis in neoadjuvant therapy response assessment is an active area of research, as these markers may provide a robust and complementary approach to genetic alterations for monitoring tumor burden and treatment efficacy (15). However, challenges remain, including the need for standardization, optimization of detection limits, and further clinical validation to establish utility across different disease stages and cancer types (9, 21) (**Table 1**).

**Table 1:** Comprehensive comparison of ctDNA detection technologies in the neoadjuvant setting.

Category	Technology type	Representative methods	Core principle	Sensitivity (LOD)	Specificity	Advantages	Limitations	Optimal clinical scenario	Turnaround time	Cost level	Key technical notes
Tumor-informed approaches	Personalized mutation tracking	WES/WGS-informed panels (e.g., RaDaR, NeXT Personal, Invitae PCM™)	Tumor tissue sequencing → patient-specific mutation panel → plasma tracking	Ultra-high (10^-5^–10^-6^; ~0.001–0.01%)	Very high (>99.9%)	- Highest sensitivity for MRD detection - Low false positives (CHIP filtered) - Suitable for longitudinal monitoring	- Requires tumor tissue - Long setup time - Expensive - Limited for rapid decisions	- MRD detection (post-op) - Recurrence prediction - Longitudinal monitoring	Long (2–4 weeks initial setup)	High	Requires matched tumor-normal sequencing; panel size affects sensitivity
Tumor-agnostic approaches	Fixed gene panel NGS	CAPP-Seq, Guardant360, FoundationOne Liquid	Predefined cancer gene panels applied directly to cfDNA	Moderate (10^-^³–10^-4^; ~0.1–0.01%)	Moderate–high	- No tumor tissue required - Faster implementation - Broad mutation detection	- Lower sensitivity in low tumor burden - CHIP interference - Background noise	- Baseline genotyping - Treatment selection - Advanced disease profiling	Medium (7–14 days)	Medium–high	Requires bioinformatic CHIP filtering; panel size vs depth trade-off
Digital PCR-based methods	Targeted mutation detection	ddPCR, BEAMing	Partitioned amplification and digital counting of mutant alleles	High (10^-4^–10^-5^; ~0.01–0.001%)	Very high	- High precision quantification - Low cost per assay - Fast turnaround	- Limited to known mutations - Low multiplexing capability	- Known driver mutation monitoring (e.g., EGFR, KRAS) - Early response tracking	Short (1–3 days)	Low–medium	Ideal for serial monitoring; requires prior mutation knowledge
Methylation-based assays	Epigenetic profiling	MS-ddPCR, MeD-Seq, cfDNA methylome assays (e.g., Northstar Response™)	Detect tumor-specific DNA methylation patterns	High (~10^-4^–10^-5^)	High	- Tissue-of-origin information - Early detection potential - Less affected by mutation heterogeneity	- Complex data interpretation - Standardization lacking	- Early cancer detection - MRD detection - Tumor origin inference	Medium	Medium–high	Multi-marker panels improve robustness
Fragmentomics-based approaches	cfDNA fragment analysis	Fragment size profiling, end motif analysis	Tumor DNA shows shorter fragment size and specific patterns	Emerging (variable)	Moderate	- No mutation dependence - Complements genomic assays	- Lower specificity alone - Still under validation	- MRD adjunct - Multi-omics integration	Medium	Medium	Often combined with methylation/genomic data
Ultra-sensitive emerging technologies	Nanotechnology/single-molecule detection	SIMUL, AuNP-DNA walker, nanopore-based detection	Signal amplification at single-molecule level	Ultra-high (<10^-5^)	High	- Extremely high sensitivity - Potential for ultra-low ctDNA detection	- Limited clinical validation - Complex setup	- Early-stage detection - Research applications	Variable	High (currently)	Not yet standardized
Multi-omics integrated platforms	Combined genomic + epigenomic + proteomic	ctDNA + CTC + exosome + protein markers	Integrative biomarker modeling	Highest (theoretical)	Highest	- Overcomes single-modality limitations - Improves sensitivity & specificity	- Complex workflow - High cost - Data integration challenges	- Precision oncology - Adaptive therapy trials	Long	Very high	Future direction; requires AI-based integration

### 2.2 Sensitivity and specificity challenges and countermeasures

The detection of ctDNA in the context of neoadjuvant therapy for solid tumors is fundamentally constrained by two primary analytical challenges: achieving sufficient sensitivity to detect extremely low-frequency variants and ensuring high specificity to avoid false-positive results. During the initial phase of neoadjuvant treatment or following successful therapy when tumor burden is minimal, ctDNA levels can be exceptionally low, often falling below 0.01% variant allele frequency (VAF) (22, 23). This presents a formidable technical hurdle, as conventional sequencing and PCR-based methods are limited by background noise, sequencing errors, and the vast excess of wild-type cell-free DNA (22). Furthermore, achieving high specificity is complicated by biological confounders, most notably clonal hematopoiesis of indeterminate potential (CHIP), where somatic mutations originating from hematopoietic cells can be detected in plasma and mistaken for tumor-derived variants, leading to false-positive results (8). This issue is particularly pronounced in assays analyzing fixed panels of common cancer driver genes, as these genes are also frequently mutated in CHIP (24). The clinical consequence of these limitations is a potential reduction in both the sensitivity for detecting MRD and the specificity required for reliable prognostic stratification and early relapse prediction.

To overcome the sensitivity barrier, advanced technological strategies focusing on error suppression and deep sequencing are essential. Employing ultra-deep next-generation sequencing (NGS) at high coverage depths (e.g., >10,000x) is a foundational approach to improve the signal-to-noise ratio for low-frequency mutations (25). The integration of unique molecular identifiers (UMIs) or molecular barcodes is critical for distinguishing true somatic variants from PCR or sequencing artifacts by enabling the tracking of individual DNA molecules (4, 25). For instance, the SIMUL technique utilizes single-molecule multicolor imaging following unbiased preamplification to achieve highly sensitive and specific detection of multiple low-frequency single-nucleotide variants, even in a 10,000-fold excess of wild-type DNA (22). Similarly, novel nanotechnology-based platforms, such as the Exonuclease III-assisted gold nanoparticle-conjugated DNA walker (Au-3D walkers), exploit enzymatic amplification mechanisms on nanoparticle surfaces to achieve ultrasensitive ctDNA detection without requiring complex enzyme-specific sequence designs (23). Optimizing pre-analytical steps is equally important; using specialized blood collection tubes (e.g., Streck BCT) and efficient cfDNA extraction protocols that maximize the recovery of short DNA fragments (~170 bp) characteristic of ctDNA can significantly improve input material quality and subsequent detection rates (26, 27). Furthermore, increasing the analytical breadth by targeting a larger number of patient-specific mutations, as seen in whole-genome sequencing (WGS) or whole-exome sequencing (WES)-informed assays, enhances the probability of detecting ctDNA molecules, thereby improving sensitivity, especially when plasma volume is limited (4, 28).

**Figure 1:**
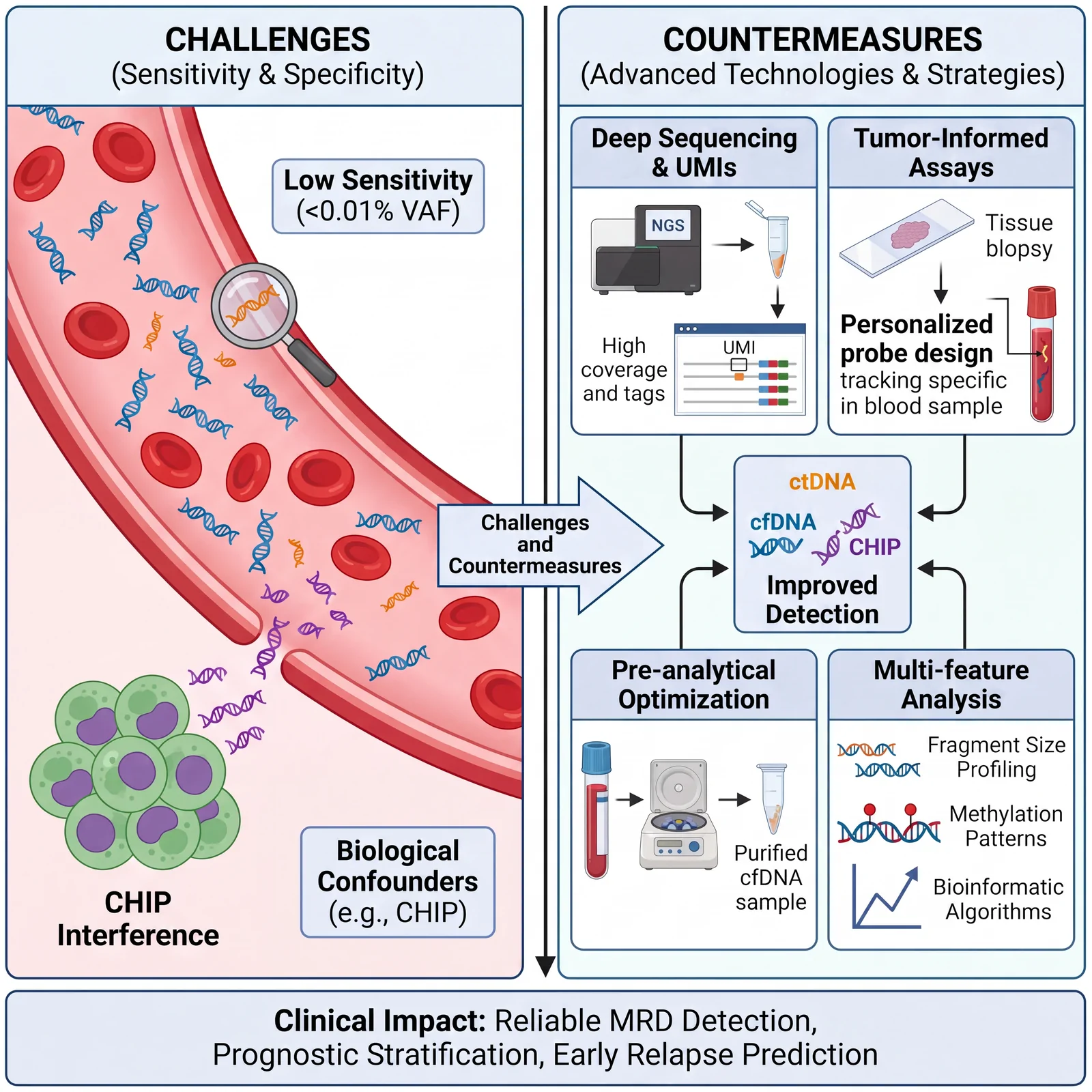
Overview of ctDNA detection technologies and analytical challenges in neoadjuvant therapy. Tumor-informed, tumor-agnostic, and methylation-based platforms are summarized together with the principal analytical challenges (low variant allele frequency, sequencing error, clonal hematopoiesis) and corresponding optimization strategies.

Enhancing specificity to discriminate tumor-derived ctDNA from noise, particularly CHIP-derived variants, requires sophisticated bioinformatic and assay design strategies. Tumor-informed approaches, where a patient’s specific mutational profile is first identified from tumor tissue and then tracked in plasma, offer superior specificity compared to tumor-agnostic panels that screen fixed gene sets (4, 29). However, tissue-informed methods can be limited by tissue availability and turnaround time (30). To address the CHIP challenge, bioinformatic algorithms can leverage multiple features of ctDNA beyond the mutation itself. These include analyzing the fragment size profile, as tumor-derived ctDNA fragments are typically shorter than those from non-malignant cells; examining methylation patterns, as tumor DNA exhibits distinct hypermethylation or hypomethylation signatures; and considering the genomic context and mutation allele frequency patterns (24, 31). For example, assays focusing on universally methylated regions in cancer, such as ZSCAN12 and OXT in endometrial carcinoma, can provide highly specific detection of methylated ctDNA (meth-ctDNA) with minimal interference from CHIP or other non-tumor sources (32). Combining multiple analyte types or features also improves specificity. A comprehensive tumor-agnostic evaluation demonstrated that a methylation-based MRD (mMRD) assay outperformed a genomic mutation-based (gMRD) approach, as the latter was significantly confounded by CHIP-derived pathogenic mutations in common cancer driver genes (24). Standardization of pre-analytical and analytical workflows across laboratories is another critical countermeasure to ensure reproducible and reliable specificity (33, 34). Finally, the clinical interpretation of ctDNA results must be contextual, considering factors such as the assay’s limit of detection, the patient’s clinical and imaging findings, and the timing of sample collection relative to therapy, to accurately distinguish true molecular residual disease from analytical artifacts or biologically irrelevant findings (8, 35) (**Figure 1**).

In synthesizing this technical landscape, three points deserve emphasis. First, the choice between tumor-informed and tumor-agnostic strategies is a genuine trade-off—sensitivity and specificity versus cost, tissue dependence, and turnaround time—rather than a question with a single correct answer, and the optimal platform differs between response monitoring during therapy and ultrasensitive MRD detection. Second, much of the apparent inconsistency among the clinical studies discussed later in this review is attributable less to biology than to assay heterogeneity: differences in limits of detection, positivity thresholds, and handling of clonal hematopoiesis can move the same patient across the ctDNA-positive/negative boundary. Third, head-to-head comparisons of platforms at clinically relevant decision points remain scarce, and defining the minimal analytical performance required to support neoadjuvant treatment decisions is an unresolved priority.

## 3 Baseline ctDNA characteristics before neoadjuvant therapy: prognostic and predictive value

### 3.1 Baseline ctDNA detection rate and concentration as prognostic biomarkers

The detection of ctDNA prior to the initiation of neoadjuvant therapy serves as a powerful, non-invasive prognostic biomarker across various solid tumors, including breast cancer, colorectal cancer (CRC), and NSCLC. The presence of ctDNA at baseline is consistently and independently associated with more advanced disease characteristics and poorer clinical outcomes. In operable breast cancer, a systematic review and meta-analysis demonstrated that ctDNA detection at baseline was significantly associated with worse disease-free survival (DFS) and overall survival (OS) (36). This association extends to other malignancies; in muscle-invasive bladder cancer (MIBC), detectable ctDNA before radical cystectomy was predictive of nodal involvement, locally advanced disease, and subsequent recurrence (37). Similarly, in resectable NSCLC, the presence of ctDNA at clinical diagnosis is linked to higher pathological tumor stage, nodal metastasis, and features like solid adenocarcinoma pattern and tumor necrosis, which collectively indicate a greater tumor burden and more aggressive biology (38). The prognostic implication is not limited to common carcinomas; in advanced urothelial cancer, a high circulating tumor DNA tumor fraction (ctDNA TF ≥1%) at a single time point was associated with poor prognostic clinical features and significantly reduced real-world progression-free survival (39). Furthermore, in gastrointestinal malignancies such as hepatocellular carcinoma (HCC), an elevated ctDNA-based score was strongly correlated with aggressive tumor features including larger tumor size, macrovascular invasion, and advanced TNM stage, and served as an independent predictor of worse overall survival (40). These findings underscore that the mere detectability of ctDNA before treatment is a robust indicator of unfavorable tumor biology and higher metastatic potential.

**Figure 2:**
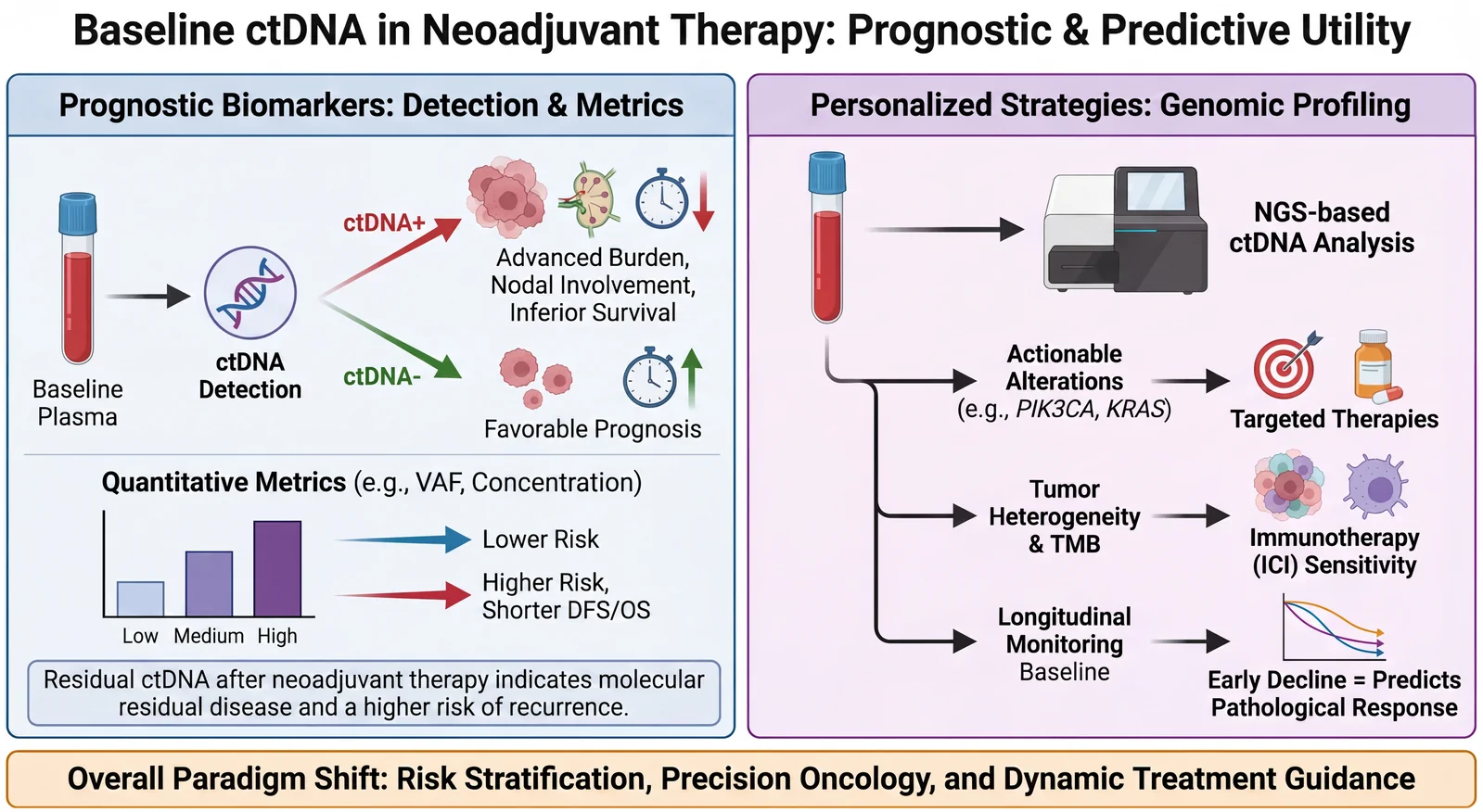
Prognostic and predictive value of baseline ctDNA before neoadjuvant therapy. Detectable and higher-level baseline ctDNA indicates greater tumor burden and worse survival, while baseline genomic profiling identifies actionable alterations for individualized treatment selection.

Beyond simple detection, the quantitative measurement of ctDNA, expressed as VAF, variant allele fraction, or concentration (e.g., copies per milliliter of plasma), provides a more granular prognostic stratification and has been validated as an independent predictor of both disease-free and overall survival. This quantitative relationship allows for the identification of high-risk patients even before therapeutic intervention begins. In colorectal cancer, studies have shown that higher baseline ctDNA levels are prognostic for shorter survival (41). This is corroborated by research in metastatic CRC, where high ctDNA levels (≥5%) before treatment with trifluridine/tipiracil were associated with inferior overall survival (42). In pancreatic ductal adenocarcinoma (PDAC), the detection of mutant KRAS (mKRAS) ctDNA in plasma at diagnosis in patients with ostensibly localized disease identified those at high risk for occult metastatic progression and worse survival outcomes (43). Similarly, in ovarian cancer, preoperative plasma ctDNA levels, measured in mutant copies per milliliter, increased with higher disease stage, and a concentration of more than 10 mutant copies/ml was significantly associated with worse overall survival (44). The prognostic value of ctDNA concentration is also evident in specific mutational contexts, as illustrated by two independent HCC cohorts in which *TERT* promoter mutations in ctDNA, particularly at high fractional abundance, were associated with shorter survival, advanced TNM stage, and vascular invasion (45, 46). In the context of neoadjuvant therapy for HER2-positive early breast cancer, patients who achieved a pCR but had detectable ctDNA after neoadjuvant therapy experienced a significantly shorter recurrence-free survival compared to pCR patients with undetectable ctDNA, highlighting that residual molecular disease, even in the absence of pathological evidence, portends a poor prognosis (47). Quantitative features beyond absolute level, such as allele-frequency heterogeneity in NSCLC, have likewise been linked to unfavorable survival across treatment modalities (48). These collective data firmly establish that both the qualitative detection and the quantitative concentration of baseline ctDNA are indispensable tools for risk stratification, enabling clinicians to identify patients with a high likelihood of recurrence and poor survival who may benefit from more intensive or novel treatment strategies in the neoadjuvant and adjuvant settings (**Figure 2**).

### 3.2 Baseline ctDNA gene profile guides individualized treatment strategies

The non-invasive profiling of ctDNA at baseline has emerged as a cornerstone for guiding personalized neoadjuvant treatment strategies in solid tumors. By capturing the genetic landscape of the tumor from a simple blood draw, ctDNA analysis can reveal actionable driver mutations that are critical for selecting targeted therapies. For instance, in breast cancer, the detection of mutations in genes such as *PIK3CA* or *ESR1* can inform the use of specific inhibitors, like PI3K inhibitors, in the neoadjuvant setting (49). Similarly, in NSCLC, identifying *EGFR* or *KRAS* mutations from plasma ctDNA provides a rationale for employing EGFR tyrosine kinase inhibitors (TKIs) or exploring other targeted approaches, thereby offering a treatment path when tissue biopsy is infeasible or insufficient (50). This approach is supported by clinical evidence showing that ctDNA-based genomic profiling can identify clinically actionable alterations with high concordance to tissue testing, enabling therapy selection even in advanced stages (51). The utility extends beyond single-gene assays; comprehensive genomic profiling via NGS of ctDNA can simultaneously assess a broad panel of cancer-related genes, uncovering alterations that qualify patients for targeted therapies or clinical trials (52). This is particularly valuable in neoadjuvant therapy, where understanding the tumor’s molecular drivers at diagnosis can help design a more effective preoperative regimen aimed at reducing tumor burden and improving surgical outcomes.

Beyond identifying individual driver mutations, baseline ctDNA analysis provides a comprehensive view of the tumor’s molecular subtype and clonal architecture, which is essential for predicting response to conventional chemotherapy or immunotherapy. The molecular heterogeneity captured by ctDNA, which reflects DNA shed from all tumor sites, can reveal subclonal populations that might be missed by a single tissue biopsy (53). This comprehensive profile allows for the assessment of biomarkers such as tumor mutational burden (TMB). High TMB, as measured from ctDNA (bTMB), has been associated with increased sensitivity to immune checkpoint inhibitors (ICIs) in various cancers, including lung cancer and urothelial carcinoma (52, 54). In the context of neoadjuvant immunotherapy for cancers like muscle-invasive bladder cancer or melanoma, baseline ctDNA levels and specific genomic features can stratify patients likely to benefit. For example, in melanoma patients receiving neoadjuvant ipilimumab and nivolumab, baseline ctDNA detectability was common, and its clearance post-treatment correlated strongly with improved outcomes (55). Furthermore, the integration of ctDNA profiling with other omics data can refine molecular subtyping. In breast cancer, ctDNA analysis not only detects *PIK3CA* or *TP53* mutations but can also inform on the tumor’s endocrine resistance profile or homologous recombination deficiency status, which could guide the use of PARP inhibitors in selected cases (56, 57). This ability to reflect both the dominant clone and minor subclones at baseline is crucial for anticipating *de novo* or early adaptive resistance to neoadjuvant chemotherapy or targeted agents, allowing for the potential combination or sequential use of therapies to overcome resistance (49).

The clinical implementation of baseline ctDNA to guide neoadjuvant therapy is increasingly supported by real-world evidence and technological advancements. Assays such as tagged-amplicon deep sequencing (TAm-Seq), cancer personalized profiling by deep sequencing (CAPP-Seq), and digital PCR platforms enable sensitive and specific detection of mutations from plasma, even at low allele fractions (58). Studies demonstrate that ctDNA profiling can identify patients with potentially actionable alterations (e.g., Tier I-II according to ESCAT classification) in a significant proportion of cases, directly impacting therapeutic decisions (52). For example, in a cohort of Italian lung cancer patients, liquid biopsy NGS identified actionable alterations in over a quarter of samples, facilitating personalized therapy and clinical trial enrollment (52). In the neoadjuvant setting for rectal cancer, baseline ctDNA detection has been associated with more advanced clinical stage and could potentially help in stratifying patients for intensified neoadjuvant regimens (59). Moreover, the dynamic and real-time nature of ctDNA analysis means that the baseline profile serves as a reference for subsequent monitoring during therapy, enabling early assessment of molecular response. This is exemplified in trials where a decline in ctDNA levels early during neoadjuvant treatment for breast or bladder cancer predicted pathological response and survival (60, 61). Therefore, incorporating baseline ctDNA genotyping into the initial workup of patients eligible for neoadjuvant therapy represents a paradigm shift towards truly personalized oncology. It allows clinicians to select the most appropriate targeted or immunotherapeutic agents upfront, tailor combination strategies based on the tumor’s genetic makeup, and establish a biomarker for longitudinal monitoring, ultimately aiming to improve pathological complete response rates and long-term outcomes (8, 62) (**Figure 2**).

Critically appraised, the prognostic value of baseline ctDNA detection is among the most consistently replicated findings in this field, but several caveats temper its interpretation. Detection rates vary widely across tumor types and stages, so a negative baseline result often reflects limited shedding rather than indolent disease. Because ctDNA levels correlate strongly with tumor burden, part of their prognostic signal overlaps with conventional staging, and the independent increment over established clinicopathological models has not been quantified consistently across studies. Finally, whereas baseline genotyping to select targeted therapy is standard practice in advanced disease, its use to direct neoadjuvant regimens remains investigational and should be clearly distinguished from the validated prognostic role.

## 4 Dynamic changes of early ctDNA in neoadjuvant therapy: real-time efficacy assessment

### 4.1 ctDNA clearance at treatment initiation as an early response marker

A substantial body of evidence demonstrates that the rapid clearance of ctDNA during the initial cycles of neoadjuvant therapy serves as a powerful early molecular marker of treatment efficacy, often preceding and outperforming traditional radiological assessments. Studies across various solid tumors, including breast, colorectal, and lung cancers, consistently show that patients who achieve ctDNA clearance—defined as the disappearance of detectable tumor-derived mutations in plasma—within the first few weeks of treatment have significantly higher rates of pCR and superior long-term outcomes compared to those with persistent ctDNA. In early-stage breast cancer, research indicates that ctDNA clearance as early as week 3 of neoadjuvant therapy predicts favorable responses across different receptor subtypes, including to immunotherapies (61). Patients with neoadjuvant therapy-resistant tumors (characterized by high residual cancer burden) who clear their ctDNA exhibit a significantly decreased risk of metastatic recurrence (61). Similarly, in triple-negative breast cancer (TNBC), clearance of ctDNA by the mid-point of neoadjuvant chemotherapy is strongly associated with achieving pCR (63). The prognostic power of this early molecular response is further highlighted in colorectal cancer. For patients with colorectal liver metastases (CRLM) receiving neoadjuvant therapy, a dynamic change in ctDNA mean variant allele frequency of 50% or more after just one cycle is significantly correlated with better treatment responses and serves as an independent predictor of improved recurrence-free survival, outperforming conventional tumor markers like CEA and CA19–9 (64). This early ctDNA dynamic has been shown to be a superior predictor of both radiologic and pathologic response (64).

The clinical utility of early ctDNA clearance lies in its ability to provide a real-time, non-invasive snapshot of tumor burden dynamics, offering a critical window for therapeutic decision-making weeks before standard imaging can confirm a response. The short half-life of ctDNA allows it to reflect tumor cell death almost in real-time, transforming the traditional “black box” period of neoadjuvant therapy into an era of dynamic monitoring (1). This capability is particularly valuable for identifying non-responders early. For instance, in advanced NSCLC treated with EGFR tyrosine kinase inhibitors, complete clearance of EGFR mutations in ctDNA by 8 weeks is highly and significantly predictive of both prolonged progression-free survival and overall survival, outperforming RECIST response criteria for predicting long-term benefit (65). Conversely, the persistence of ctDNA midway through neoadjuvant therapy for breast cancer is strongly associated with disease recurrence, especially in HER2-negative disease, and enhances the prognostic accuracy of residual cancer burden scores (66). This early molecular stratification enables the potential for timely intervention. Patients showing rapid ctDNA clearance may be candidates for treatment de-escalation strategies to minimize toxicity, while those with persistent ctDNA could be considered for therapy intensification or a switch to alternative regimens before clinical progression becomes evident (1). The predictive value extends to immunotherapy settings as well. A systematic review and meta-analysis focusing on neoadjuvant immune checkpoint inhibitors in solid tumors found that the lack of ctDNA clearance may identify patients unlikely to achieve a pCR, although the confirmatory power of clearance is currently limited by assay variability (67). Nonetheless, in diverse solid tumors treated with combination immunotherapy, ctDNA clearance at any time through week 8 has been shown to identify complete responders with a median lead time of 11.5 months ahead of radiographic imaging (68). Thus, the integration of early ctDNA dynamics into clinical practice promises to shift the paradigm from static, post-treatment assessment to a dynamic, response-adapted approach, maximizing therapeutic efficacy and personalizing care in the neoadjuvant setting.

### 4.2 ctDNA kinetics patterns and their correlation with pathological response

The dynamic changes in ctDNA levels during neoadjuvant therapy, known as ctDNA kinetics, have emerged as a powerful, real-time biomarker for predicting pathological response. These kinetic patterns can be broadly categorized into distinct profiles that correlate strongly with the degree of tumor regression observed in the surgical specimen. The most favorable pattern is the “rapid clearance” type, where ctDNA becomes undetectable early in the treatment course. This pattern is strongly associated with a high rate of pCR or major pathological response (MPR). For instance, in TNBC, patients who achieved ctDNA clearance by the midpoint of neoadjuvant chemotherapy (mid-NAC) had a significantly higher rate of pCR (63). Similarly, in resectable NSCLC treated with neoadjuvant chemoimmunotherapy, ctDNA clearance before surgery was consistently linked to higher rates of MPR (69). This association extends to other malignancies; in a phase II trial of neoadjuvant camrelizumab and apatinib for NSCLC, perioperative ctDNA clearance was significantly associated with improved event-free survival (70). The predictive value of early clearance is so pronounced that in locally advanced rectal cancer (LARC), a “low-risk” group defined by early ctDNA clearance had a dramatically lower rate of poor pathological response compared to a “high-risk” group (71). This rapid clearance reflects a profound and systemic antitumor effect, effectively eradicating micrometastatic disease and translating to superior long-term outcomes (1).

In contrast, a “slow decline” pattern, characterized by a gradual reduction in ctDNA levels without achieving complete clearance, typically corresponds to a partial pathological response. Patients exhibiting this kinetic profile often have residual viable tumor at surgery, indicating a suboptimal but present treatment effect. Studies across various cancers show that the rate of ctDNA clearance during therapy correlates inversely with the amount of residual disease. For example, in LARC patients undergoing neoadjuvant chemoradiotherapy, the ctDNA clearance rate showed a significant decreasing trend across groups with worsening pathological tumor regression grades (72). This pattern suggests that while the treatment is exerting pressure on the tumor, it may not be sufficient to eliminate all clonal populations. The clinical implication is that these patients, despite not achieving a pCR, may still derive significant benefit from the neoadjuvant regimen, though they remain at a higher risk of recurrence compared to rapid clearers. The integration of such ctDNA dynamics with other biomarkers, like peripheral blood immune cell counts, can further refine the understanding of the ongoing antitumor immune response in this intermediate group (73).

**Figure 3:**
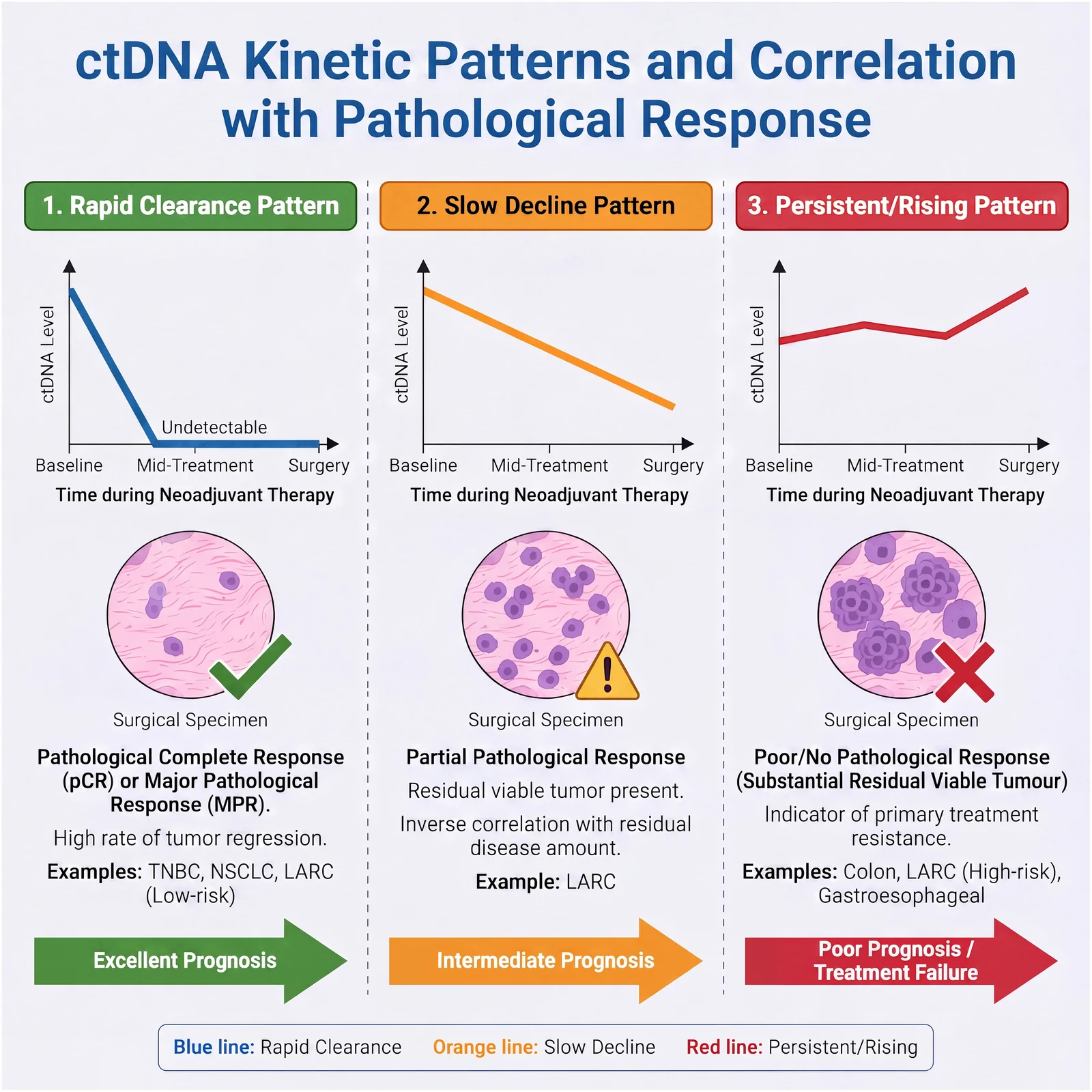
Early ctDNA kinetic patterns during neoadjuvant therapy. Rapid clearance predicts pathological complete response and favorable outcomes; gradual decline indicates partial response; persistent or rising ctDNA signals treatment resistance and high recurrence risk.

The most concerning kinetic pattern is “persistent positivity or rising” ctDNA levels during therapy. This profile is highly indicative of primary treatment resistance or outright disease progression and is almost invariably associated with the absence of pathological response (e.g., pathological non-response or stable/progressive disease). Persistent detectable ctDNA after neoadjuvant therapy is a strong predictor of residual viable tumor at resection. A systematic review in non-metastatic colon cancer found that persistent ctDNA strongly predicted residual tumor, and no study reported a pCR in patients with persistently positive ctDNA (74). In LARC, patients classified as “high-risk” based on delayed/no clearance or recurred positivity of ctDNA had a very high rate of poor pathological response, and none achieved a major pathological response (71). Similarly, in gastroesophageal cancer treated with neoadjuvant immunotherapy, patients who failed to clear ctDNA post-induction had significantly worse recurrence-free and overall survival (75). This pattern underscores the utility of ctDNA as an early indicator of therapeutic failure, potentially allowing for timely intervention, such as treatment intensification or switch to an alternative regimen, long before clinical or radiographic progression becomes apparent (1). The biological underpinning likely involves the survival and expansion of resistant tumor clones, which continue to shed DNA into the bloodstream, making ctDNA a critical tool for identifying patients who are not benefiting from standard neoadjuvant approaches (**Figure 3**).

Across these studies a consistent asymmetry emerges: persistent ctDNA reliably identifies patients who will not achieve pCR, whereas clearance does not guarantee pCR, because low-shedding tumors and assay sensitivity limits generate biologically “false-negative” results. The I-SPY 2 observation that early clearance is predictive in triple-negative but not in hormone receptor-positive/HER2-negative disease illustrates that kinetic thresholds are unlikely to be transferable across subtypes, let alone across tumor types. Divergent findings between studies are further explained by non-standardized sampling timepoints and differing definitions of “clearance”. The central unanswered question is therefore not whether early kinetics carry prognostic information—they clearly do—but whether modifying treatment on the basis of them improves outcomes, which no completed trial has yet demonstrated.

## 5 ctDNA monitoring during neoadjuvant therapy: guiding treatment intensity and duration

### 5.1 The value of mid-treatment ctDNA status for treatment plan adjustment

The dynamic assessment of ctDNA during neoadjuvant therapy provides a critical opportunity to adapt treatment strategies, moving beyond the traditional “black box” period where patients may receive ineffective and toxic therapies (1). For patients who achieve ctDNA clearance early or midway through treatment, there is a growing body of evidence suggesting a favorable prognosis, which raises the question of potential overtreatment with the full standard regimen. As detailed in Section 3, rapid molecular responses—such as week-3 clearance in breast cancer (61) or an early decline in variant allele frequency in colorectal liver metastases (64)—are detectable long before radiological assessment and provide a strong rationale for exploring treatment de-escalation strategies, such as shortening the duration of chemotherapy or reducing its intensity, to minimize treatment-related toxicity without compromising oncological outcomes (1). The concept is further supported by data showing that patients with early-stage triple-negative breast cancer who clear ctDNA after neoadjuvant chemotherapy have a markedly lower risk of relapse, suggesting that a subset of patients with excellent molecular responses may be candidates for less intensive adjuvant approaches (76). It must be stressed, however, that no completed randomized trial has yet demonstrated the safety of de-escalation triggered by mid-treatment ctDNA negativity; given the imperfect sensitivity of current assays and the existence of low-shedding tumors, such strategies should for now be confined to prospective clinical trials (1).

Conversely, the persistence of ctDNA during neoadjuvant therapy is a powerful indicator of suboptimal response and high risk for disease recurrence, providing a molecular basis for treatment intensification in clinical trials (1). Across multiple solid tumors, including breast, rectal, and gastric cancers, patients with detectable ctDNA midway through or at the end of neoadjuvant treatment have a significantly worse prognosis. In breast cancer, persistent ctDNA detection midway through neoadjuvant therapy is strongly associated with disease recurrence, particularly in HER2-negative disease, and enhances the prognostic accuracy of traditional metrics like the residual cancer burden score (66). For patients with locally advanced rectal cancer undergoing total neoadjuvant therapy, a positive ctDNA result post-treatment, while not perfectly sensitive, has a very high positive predictive value for residual disease, identifying a group at extreme risk for recurrence who are unlikely to be candidates for non-operative management and may benefit from immediate surgical resection or alternative systemic strategies (77, 78). This molecular evidence of residual disease creates a compelling rationale for clinical trials investigating therapy intensification. Potential strategies include adding novel agents to the existing regimen, switching to a different chemotherapy backbone, incorporating radiotherapy in cancers where it is not standard, or extending the duration of treatment (1). For instance, in locally advanced rectal cancer, dynamic ctDNA monitoring has been shown to identify patients at high risk for chemotherapy failure as early as two cycles into treatment, forming a basis for adaptive trial designs where treatment could be intensified in real-time for those with unfavorable ctDNA dynamics (71). The utility of ctDNA for guiding such escalations is also being explored in the context of immunotherapy, where early ctDNA dynamics can predict long-term clinical outcomes, potentially allowing for the early addition of combination therapies in non-responders (68). Thus, mid-treatment ctDNA status serves as a pivotal decision point, enabling a shift from a one-size-fits-all approach to a response-adapted, personalized treatment paradigm in the neoadjuvant setting. To date, however, ctDNA-adaptive neoadjuvant strategies remain hypothesis-generating: they are supported by consistent prognostic associations, but not yet by randomized evidence that acting on ctDNA improves outcomes.

### 5.2 Clonal evolution and early detection of resistance mechanisms

**Figure 4:**
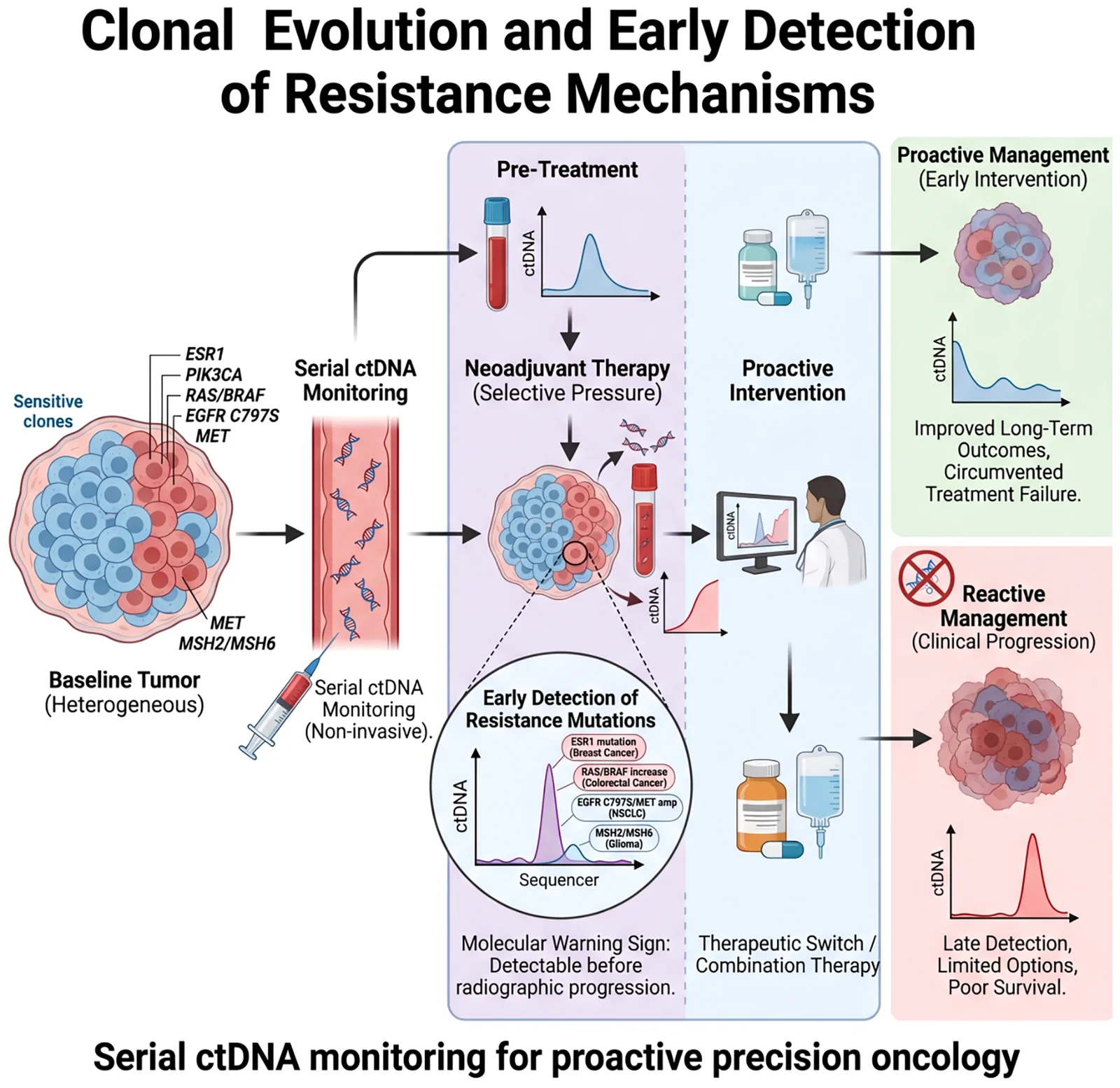
ctDNA-guided treatment adaptation during neoadjuvant therapy. Early clearance supports investigation of de-escalation, persistent ctDNA provides a rationale for intensification, and serial profiling captures clonal evolution and emerging resistance mutations.

Serial ctDNA monitoring provides a dynamic, non-invasive window into the clonal evolution of tumors under the selective pressure of neoadjuvant therapy, enabling the early detection of acquired resistance mechanisms that would otherwise remain occult until clinical or radiographic progression. This capability is particularly transformative in the context of breast cancer, where the emergence of mutations in the estrogen receptor gene *ESR1* is a well-characterized mechanism of resistance to endocrine therapies, including those used in the neoadjuvant setting for hormone receptor-positive disease (79). The analysis of ctDNA allows for the repeated sampling of the tumor’s genetic landscape, capturing the temporal heterogeneity and the rise of resistant subclones that may not be represented in a single, static tissue biopsy (80). For instance, in early-stage breast cancer patients, ctDNA analysis has revealed a broader mutation spectrum compared to tumor tissue DNA, including the early detection of *ESR1* mutations, which are signatures of endocrine therapy resistance and metastasis susceptibility (81). This early molecular warning sign, detectable in plasma before clinical signs of progression, provides a critical opportunity for therapeutic intervention. The clinical utility of ctDNA in identifying such druggable resistance mutations, including *ESR1* and *PIK3CA*, is well-established in metastatic breast cancer and is increasingly being explored in early-stage disease to guide post-neoadjuvant strategies (82, 83). Beyond single mutations, comprehensive ctDNA profiling via NGS can track the dynamics of “trunk” mutations (surrogates of total tumor burden) and specifically monitor the relative fraction of known resistance mutations, such as those in *RAS/BRAF* pathways in colorectal cancer (84). This approach was validated in a study of metastatic colorectal cancer patients receiving first-line chemotherapy plus cetuximab, where an early increase in the relative fraction of *RAS/BRAF* mutations at cycle 3 predicted shorter progression-free survival, signifying the early expansion of a resistant clone (84). Similarly, in EGFR-mutant NSCLC treated with osimertinib, longitudinal ctDNA analysis can detect resistance mechanisms (e.g., *MET* amplification, *EGFR* C797S mutations) a median of 14 weeks before radiographic progression, creating a potential window for early treatment adjustment (85, 86). This principle extends to other malignancies; in glioma, plasma ctDNA detection of mismatch repair gene mutations (*MSH2*, *MSH6*) emerged following temozolomide treatment and was frequently observed in plasma prior to their detection in tumor tissue at recurrence surgery, indicating its role in signaling the development of chemotherapy resistance (87). The early on-treatment changes in ctDNA levels or variant allele frequency are strongly linked to clinical outcomes. In metastatic castration-resistant prostate cancer (mCRPC), patients with persistent detectable ctDNA after 4 weeks of androgen receptor pathway inhibitor (ARPI) treatment had significantly worse progression-free and overall survival, and early ctDNA clearance was predictive of durable benefit, informing the potential for early therapy switches (88, 89). This early kinetic assessment is also applicable in melanoma treated with anti-PD1 immunotherapy, where a significant increase in ctDNA levels within the first weeks of treatment (biological progression) was associated with primary resistance and a lack of clinical benefit (90). By revealing the genomic trajectory of the tumor, serial ctDNA monitoring effectively maps clonal evolution, distinguishing between clonal hematopoiesis and true tumor-derived alterations, and identifies the emergence of targetable resistance pathways (91). This early discovery of resistance mechanisms provides a precious window of opportunity to intervene preemptively—either before surgery in the neoadjuvant phase or in the adjuvant setting—by switching or combining other targeted therapies, thereby aiming to circumvent treatment failure and improve long-term outcomes (49, 56). The ability to act on this molecular information dynamically is a cornerstone of advancing precision oncology, moving from a reactive to a proactive management model for solid tumors (92, 93) (**Figure 4**).

Taken together, mid-treatment ctDNA monitoring currently offers a validated prognostic readout but an unproven decision tool. Response-adapted escalation or de-escalation and preemptive targeting of emerging resistance mutations are conceptually compelling, yet they rest on extrapolation: acting on a false-negative result risks undertreatment, clonal hematopoiesis-derived or subclonal variants may prompt unnecessary treatment changes, and the lead time gained may not translate into survival benefit. These strategies should therefore be regarded as investigational until adaptive trials with ctDNA-triggered randomization report their results.

## 6 Post-neoadjuvant therapy (preoperative) ctDNA status: predicting pathological complete response and prognosis

### 6.1 High concordance between preoperative ctDNA clearance and pathological complete response

Multiple studies have established a strong positive correlation between the clearance of ctDNA in plasma samples collected after the completion of all cycles of neoadjuvant therapy and prior to surgery, and the achievement of a pCR. The negative predictive value of ctDNA for pCR is exceptionally high. A systematic review and meta-analysis focusing on patients with solid tumors treated with neoadjuvant ICIs found that the absence of ctDNA clearance was a powerful negative predictor, with a pooled sensitivity of 0.98 for predicting pCR (67). This indicates that nearly all patients who fail to clear ctDNA do not achieve pCR. Conversely, ctDNA clearance is strongly associated with favorable outcomes. In high-risk early breast cancer patients treated with neoadjuvant chemotherapy, all patients who achieved pCR were ctDNA negative at the time of surgery (94). Similarly, in a study of patients with HER2-positive or triple-negative breast cancer, all patients who achieved pCR had undetectable ctDNA after neoadjuvant therapy (95). The prognostic power of ctDNA dynamics extends beyond a simple binary pCR outcome. Research demonstrates that rapid ctDNA clearance during treatment is a powerful predictor of both pathological response and long-term survival across breast, lung, colorectal, and other solid tumors (1). Even in patients who do not achieve pCR, clearance of ctDNA is associated with significantly improved survival outcomes, suggesting it refines risk stratification beyond traditional pathological assessment (94). For certain tumor types, such as rectal cancer, preoperative ctDNA status has been shown to be a more accurate predictor of pCR than MRI-assessed tumor regression grade (TRG). A prospective cohort study in LARC patients undergoing neoadjuvant chemoradiotherapy found that a model combining ctDNA features (including clearance) with MRI TRG significantly improved the prediction of pCR/non-pCR compared to models using MRI TRG alone (72). This highlights the complementary value of ctDNA to conventional imaging. The integration of ctDNA with other modalities is a key theme. In esophageal squamous cell carcinoma (ESCC), a model combining post-neoadjuvant immunochemotherapy ctDNA concentration with PET/CT parameters (SUVmean) demonstrated superior performance in predicting pCR compared to either modality alone (96). Furthermore, in the context of total neoadjuvant therapy for LARC, clearance of ctDNA after short-course radiotherapy was significantly associated with pCR (97). These findings collectively underscore that preoperative ctDNA clearance serves as a highly sensitive, real-time, and non-invasive biomarker that strongly correlates with the depth of pathological response, offering a more dynamic and potentially more accurate assessment than static imaging evaluations for predicting pCR in several solid malignancies.

### 6.2 Preoperative ctDNA positivity as a marker of high-risk residual disease

The detection of ctDNA in the preoperative setting, following the completion of neoadjuvant therapy, serves as a powerful biomarker for the presence of MRD and is strongly predictive of a high risk of postoperative recurrence, even when standard imaging assessments indicate a clinical or radiological response. In patients with LARC treated with total neoadjuvant therapy (TNT), studies have consistently shown that a positive ctDNA test after treatment completion is a robust indicator of residual viable tumor. For instance, in a retrospective study of LARC patients, postoperative ctDNA positivity was strongly correlated with the presence of residual disease, with high specificity and sensitivity, and was significantly associated with worse disease-free survival (98). Similarly, in ESCC patients undergoing neoadjuvant chemoradiotherapy (nCRT), the detection of ctDNA after treatment was highly effective in diagnosing residual disease, with patients who had detectable MRD post-nCRT exhibiting significantly worse survival outcomes (99). This association holds true across various solid tumors. In NSCLC, detectable ctDNA after neoadjuvant chemo-immunotherapy and before surgery is associated with a higher risk of disease recurrence (69). The prognostic power of preoperative ctDNA status is underscored by its ability to identify patients who, despite achieving a clinical complete response (cCR) or partial response on imaging, harbor occult micrometastatic disease that will ultimately lead to relapse. This is critically important in contexts like rectal cancer, where a non-operative management (NOM) strategy is considered for patients with a cCR; preoperative ctDNA positivity can flag those for whom this approach carries a high risk of failure due to undetected MRD (78). The high positive predictive value of a positive ctDNA test in this setting means that its detection is a near-certain indicator that macroscopic or microscopic disease persists, translating into a dramatically elevated risk of recurrence compared to patients who clear their ctDNA (77). Therefore, preoperative ctDNA assessment transcends the limitations of conventional imaging and pathology, providing a molecular snapshot of the systemic tumor burden and offering an early, accurate warning of impending recurrence long before it becomes clinically apparent.

**Figure 5:**
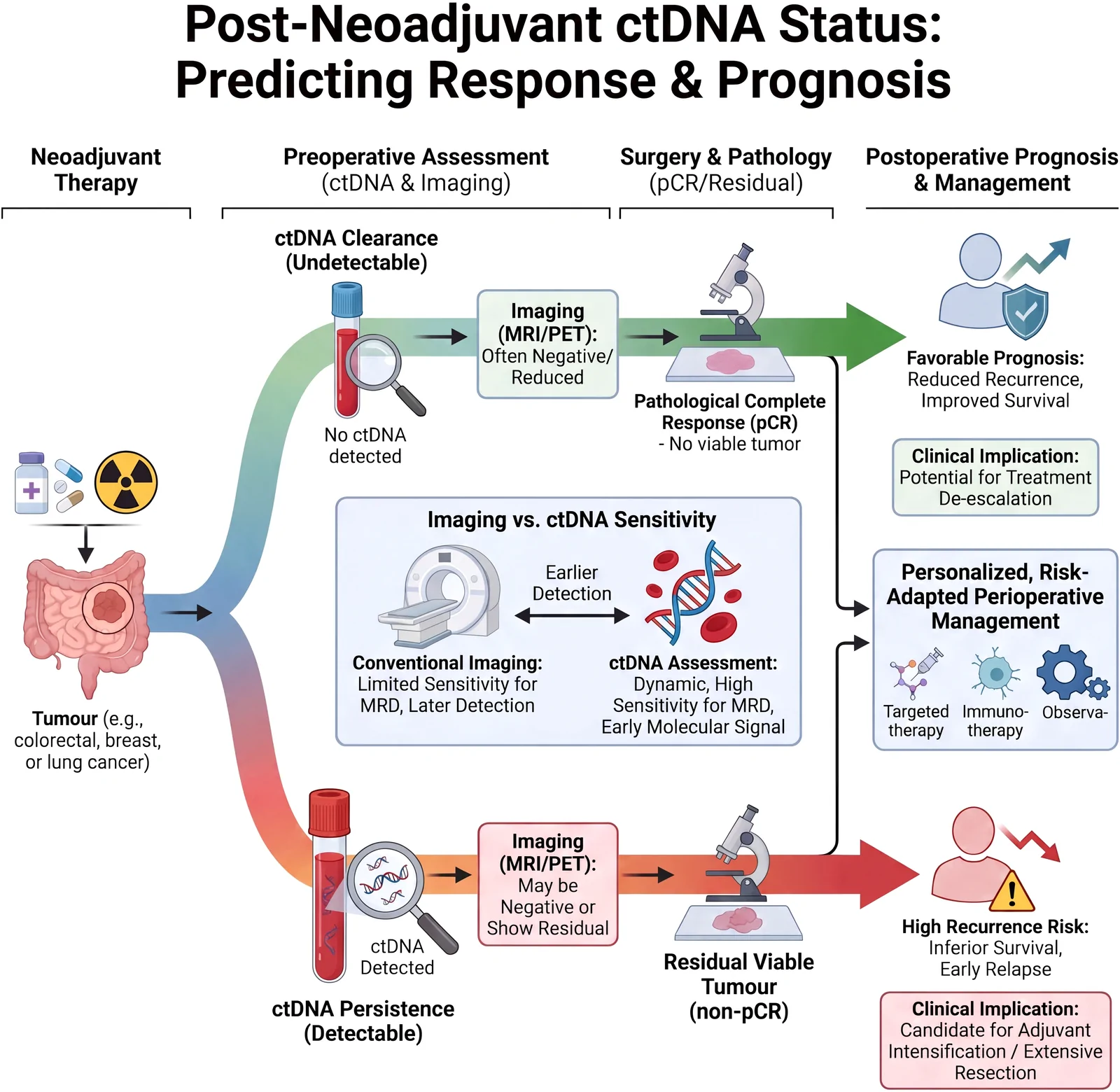
Preoperative ctDNA status after completion of neoadjuvant therapy. ctDNA clearance shows high concordance with pathological complete response, whereas detectable ctDNA indicates residual disease and high recurrence risk despite radiological response.

This critical information directly informs and refines perioperative management strategies, enabling surgeons and oncologists to tailor more aggressive interventions for patients identified as high-risk by ctDNA. For the surgical team, knowledge of persistent ctDNA could justify a more extensive resection to ensure clear margins, potentially including a wider lymphadenectomy, in an effort to eradicate all locoregional disease (78). From a medical oncology perspective, preoperative ctDNA positivity provides a compelling rationale for intensifying postoperative adjuvant therapy. Patients with detectable ctDNA after neoadjuvant treatment represent a population with a very high likelihood of harboring MRD, making them ideal candidates for adjuvant systemic therapy escalation. This could involve the use of novel agents, extended durations of treatment, or the integration of different therapeutic modalities like immunotherapy or targeted therapy based on the genomic profile of the residual disease (69, 100). In breast cancer, for example, the presence of residual disease after neoadjuvant chemotherapy is a well-established indicator of poor prognosis, and adjuvant strategies are specifically tailored for this high-risk group, such as capecitabine for triple-negative disease or trastuzumab emtansine for HER2-positive disease (101). ctDNA status adds a dynamic, molecular layer to this risk stratification. In colorectal cancer, postoperative ctDNA detection is a strong independent predictor of recurrence and is being explored to guide decisions on adjuvant chemotherapy, with the potential to escalate treatment for ctDNA-positive patients and de-escalate for those who are ctDNA-negative (102, 103). The utility of ctDNA extends to guiding the need for surgery itself in some investigational contexts. In muscle-invasive bladder cancer, detectable ctDNA after neoadjuvant therapy may predict a higher likelihood of residual tumor at cystectomy, reinforcing the decision to proceed with radical surgery (104). Conversely, while not yet standard, the strong negative predictive value of ctDNA in some settings raises the future possibility of more confidently selecting patients for organ-preservation approaches. Ultimately, by pinpointing the subset of patients with the highest risk of relapse due to MRD, preoperative ctDNA testing facilitates a shift from a one-size-fits-all postoperative plan to a risk-adapted, personalized strategy. This allows for the rational allocation of more intensive therapies to those who will benefit most, while sparing lower-risk patients from unnecessary treatment toxicity, thereby optimizing both oncologic outcomes and quality of life (1, 2) (**Figure 5**). Nevertheless, these surgery- and therapy-directing applications remain investigational; as recently emphasized for muscle-invasive bladder cancer, the prognostic value of ctDNA is robust, but its utility for personalizing treatment awaits prospective validation (105).

The practical message of the preoperative timepoint is the asymmetry of its predictive values: ctDNA positivity after neoadjuvant therapy is a highly specific indicator of residual disease, whereas negativity is insufficient to exclude it. This asymmetry currently precludes the use of ctDNA alone to select patients for organ preservation or surgical de-escalation; the most promising path forward is integration with imaging (e.g., MRI tumor regression grade or PET parameters), which has consistently outperformed either modality alone. Prospective trials in which preoperative decisions are actually modified by ctDNA status are needed before this application can enter routine practice.

## 7 Postoperative ctDNA testing: minimal residual disease assessment and recurrence risk stratification

### 7.1 Postoperative ctDNA as a potential gold standard for MRD detection

Postoperative ctDNA analysis, performed approximately 4 weeks after surgery to allow clearance of normal cell-free DNA released from surgical trauma, has emerged as one of the most sensitive tools for assessing MRD in solid tumors (106, 107). The detection of ctDNA at this landmark timepoint, signifying MRD positivity, is a powerful predictor of an extremely short DFS across various malignancies (62, 106). For instance, in CRC, a tumor-informed, hybrid-capture-based ctDNA assay detecting MRD at postoperative week 3 was strongly associated with poor DFS, with an adjusted hazard ratio of 8.40 (108). Similarly, in resected HCC, personalized MRD assessment in perisurgical plasma identified patients at high risk of recurrence, with postsurgical MRD status being the most significant risk factor in multivariate analysis (109). This prognostic power extends to other cancers, including breast cancer, where ctDNA positivity after neoadjuvant therapy completion is an independent risk factor for relapse (110, 111), and testicular cancer, where detectable ctDNA in the MRD window after orchiectomy predicted inferior event-free survival (112). The clinical validity is further underscored by studies showing that patients who convert from MRD-positive to MRD-negative status after adjuvant therapy have significantly better outcomes, highlighting ctDNA’s dynamic role in monitoring treatment efficacy (108, 113).

**Figure 6:**
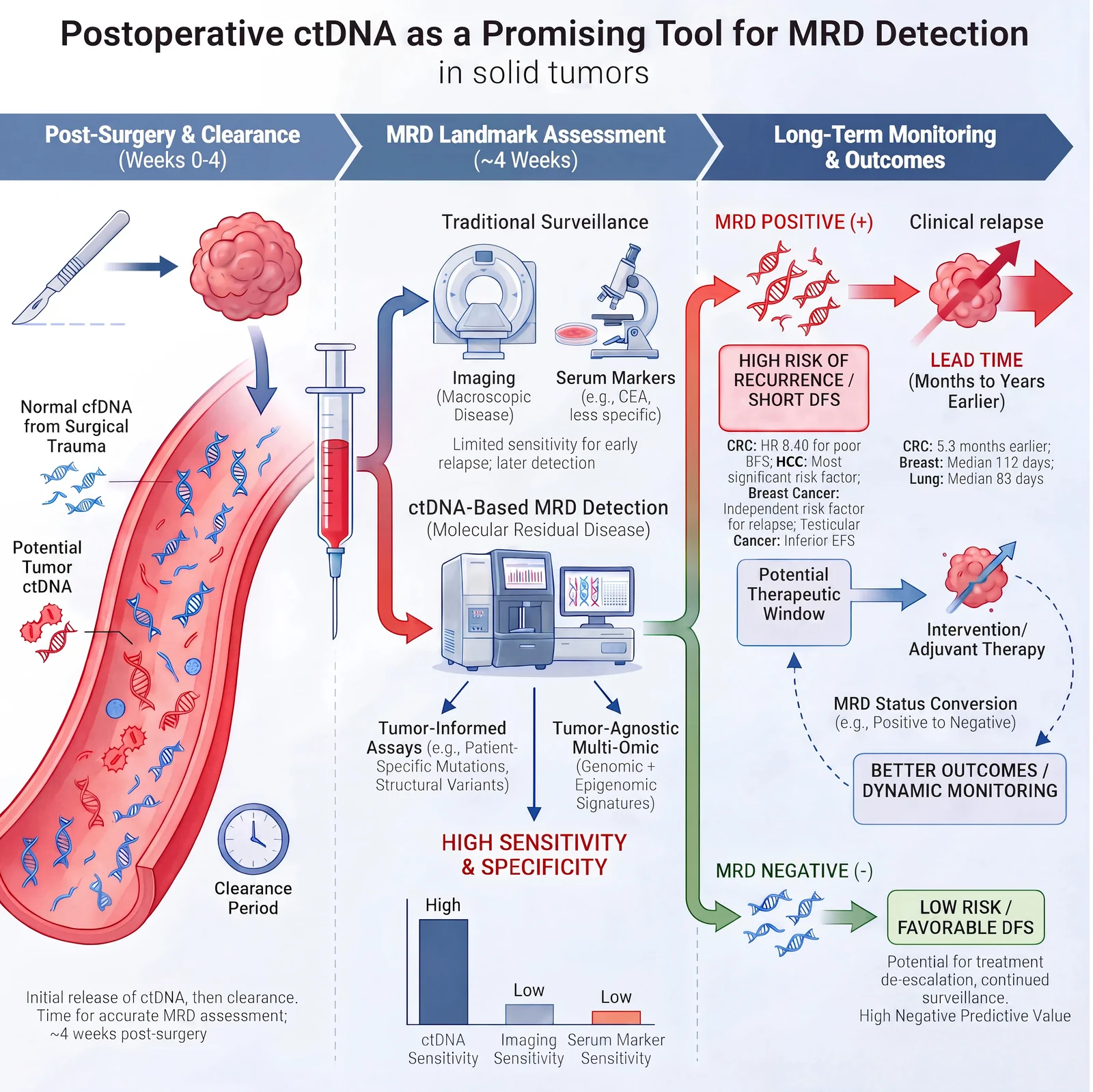
Postoperative ctDNA as a biomarker of MRD. MRD positivity after surgery predicts markedly shorter disease-free survival and precedes radiological recurrence by months.

Compared to traditional surveillance methods like imaging and serum tumor markers (e.g., CEA), ctDNA-based MRD detection provides a substantial lead time, predicting clinical recurrence months to years earlier (106, 114, 115). This advanced warning is critical as it defines a stage of molecular residual disease, offering a potential therapeutic window for intervention before overt clinical relapse (115, 116). In a pan-cancer analysis, ctDNA detection preceded radiographic evidence of recurrence by a median of 112 days in breast cancer and 83 days in lung cancer (5), and by 5.3 months in a real-world colorectal cancer analysis (117). This superior predictive capability is partly because ctDNA directly reflects the presence of viable tumor cells, whereas imaging can only detect macroscopic disease and tumor markers lack specificity and sensitivity, especially in early-stage settings (62, 118). For example, in CRC surveillance, standard serum CEA levels did not significantly predict recurrence, whereas a plasma-only ctDNA assay demonstrated a positive predictive value of 100% at the landmark timepoint (119). The sensitivity of ctDNA MRD detection, while variable depending on assay technology and tumor shedding, has been shown to be significantly higher than conventional methods for early relapse prediction (116, 120). Technological advancements, including tumor-informed assays tracking numerous patient-specific mutations or structural variants and tumor-agnostic multi-omic approaches integrating genomic and epigenomic signatures, continue to enhance detection sensitivity and specificity, solidifying ctDNA’s potential role as a transformative biomarker for postoperative monitoring and personalized adjuvant treatment strategies (5, 108, 121, 122) (**Figure 6**).

### 7.2 Risk stratification and treatment escalation/de-escalation based on postoperative MRD status

The detection of MRD through ctDNA analysis has emerged as a powerful tool for postoperative risk stratification, enabling the identification of distinct patient populations who may benefit from either intensified or reduced adjuvant therapy. This paradigm shift moves beyond traditional clinicopathologic staging to a more dynamic, biology-driven approach to adjuvant treatment decisions. The fundamental principle is that the presence of ctDNA after curative-intent surgery signifies the persistence of occult micrometastatic disease, which is a harbinger of imminent clinical recurrence. Consequently, patients are stratified into two primary groups based on their postoperative MRD status: those with detectable ctDNA (MRD-positive) and those with undetectable ctDNA (MRD-negative). This binary classification forms the basis for guiding subsequent therapeutic strategies, with the overarching goals of improving survival for high-risk patients and reducing treatment-related toxicity for those at lower risk. It should be noted at the outset that, with the exception of ctDNA-guided de-escalation of adjuvant chemotherapy in stage II colon cancer [DYNAMIC trial (123, 124)], none of the strategies discussed below has yet been validated in a completed randomized trial; postoperative MRD status is therefore best regarded, at present, as a clinically validated prognostic biomarker whose predictive utility for treatment selection is still under investigation.

- MRD-positive patients: Belonging to the ultra-high-risk population for recurrence, they are ideal candidates for clinical trials of intensified adjuvant therapy (such as additional chemotherapy, targeted therapy, or radiotherapy), aiming to eradicate MRD and improve prognosis.

Patients with detectable ctDNA after definitive surgery constitute an ultra-high-risk group with a significantly elevated likelihood of disease relapse. Across multiple solid tumor types, postoperative ctDNA positivity is a robust and independent prognostic biomarker that consistently predicts shorter disease-free and overall survival, often preceding radiographic evidence of recurrence by several months (125–127). This association holds true in diverse malignancies, including NSCLC (128, 129), CRC (130–132), MIBC (105), gastroesophageal adenocarcinoma (GEA) (133), melanoma (15), and gynecologic cancers (134). For instance, in resected stage II-III CRC, postoperative ctDNA positivity is a strong predictor of recurrence and may outperform traditional clinicopathologic risk factors (127, 135). Similarly, in NSCLC, perioperative ctDNA-based MRD detection effectively stratifies relapse risk, with MRD-positive status being a stronger predictor than conventional TNM staging (136). Given this dire prognosis, MRD-positive patients represent a critical population for intervention. The current clinical imperative is to test whether therapeutic intensification can convert this biomarker-defined high risk into improved clinical outcomes. Consequently, these patients are considered ideal candidates for clinical trials evaluating escalated adjuvant regimens. The scientific rationale is that early intervention, when the tumor burden is microscopic and potentially more susceptible to therapy, could eradicate residual clones and prevent overt metastatic disease. Ongoing trials are exploring various intensification strategies, including the addition of novel agents, extended duration of chemotherapy, or integration of targeted therapies and immunotherapies based on specific molecular alterations identified in the ctDNA (106, 137). For example, in CRC, trials are comparing standard adjuvant chemotherapy (e.g., FOLFOX/CAPEOX) against more intensive regimens like FOLFIRINOX for ctDNA-positive patients post-surgery (138). In esophageal squamous cell carcinoma, patients with detectable MRD after neoadjuvant chemoradiotherapy and surgery showed improved disease-free survival with adjuvant immunotherapy, whereas MRD-negative patients derived no benefit, highlighting a potential escalation strategy (99). The goal of these escalation trials is to translate the prognostic value of ctDNA into predictive utility, ultimately defining effective MRD-directed therapies that can clear the molecular residual disease and improve long-term survival for this vulnerable patient subset (139, 140).

- MRD-negative patients: Having a lower risk of recurrence, they may become future candidates for treatment de-escalation (such as reducing adjuvant therapy cycles or intensity, or even observation) to avoid unnecessary treatment toxicity.

Conversely, patients who achieve undetectable ctDNA status following curative-intent surgery are classified as having a low risk of recurrence. Multiple prospective studies have demonstrated that MRD-negative status is associated with excellent long-term outcomes, with disease-free survival rates often exceeding 90% at various time points (95, 141). This high negative predictive value suggests that a significant proportion of patients currently receiving standard adjuvant chemotherapy based on clinicopathologic features may be overtreated, exposing them to substantial physical, financial, and quality-of-life toxicities without deriving a meaningful survival benefit (103, 142). Therefore, MRD-negative status provides a compelling rationale for exploring treatment de-escalation strategies. The potential to safely reduce or even omit adjuvant therapy in this low-risk group represents a major opportunity to minimize overtreatment and its associated burdens. Several ongoing clinical trials are designed to test the non-inferiority of ctDNA-guided surveillance or reduced therapy compared to standard adjuvant treatment (140, 143). For instance, in the CIRCULATE-US trial for early-stage colon cancer, patients with negative postoperative ctDNA are randomized to receive either immediate standard adjuvant chemotherapy or serial ctDNA surveillance with delayed therapy only upon conversion to ctDNA positivity (138). Similarly, the TRACC Part C trial in high-risk stage II/III CRC randomizes patients to ctDNA-guided chemotherapy versus standard-of-care, with the hypothesis that de-escalation in ctDNA-negative patients is non-inferior (143). This de-escalation paradigm is also being investigated in other cancers, such as breast cancer, where patients with HER2-positive or triple-negative disease who are ctDNA-negative after neoadjuvant therapy and surgery have shown exceptional 5-year invasive disease-free survival, supporting potential de-escalation strategies (95). Analogous MRD-guided de-escalation concepts are being explored beyond solid tumors as well (144). The successful validation of such approaches would revolutionize adjuvant care by personalizing treatment intensity based on real-time molecular evidence of residual disease rather than static pathological features. It would shift the paradigm from “one-size-fits-all” adjuvant therapy to a dynamic, risk-adapted model where treatment is escalated only for those with proven molecular evidence of residual disease (MRD-positive), while sparing those without such evidence (MRD-negative) from unnecessary toxicity (2, 137, 145). This precision oncology framework promises to improve the therapeutic ratio and patient quality of life across a broad spectrum of solid tumors.

Postoperative MRD assessment is the most mature clinical application of ctDNA, and it is also where the distinction between prognostic validity and clinical utility is sharpest. Randomized evidence of utility currently exists only for de-escalation in stage II colon cancer [DYNAMIC (123, 124)], whereas the benefit of escalation in MRD-positive patients—arguably the more pressing clinical question—remains unproven, with supportive signals such as adjuvant immunotherapy in MRD-positive esophageal cancer (99) still hypothesis-generating. Unresolved issues include the discordant sensitivity of different assays at the landmark timepoint, the added value of serial over single-timepoint testing, and whether treating molecular disease earlier genuinely alters the natural history of relapse rather than merely advancing the date of its detection (105).

## 8 ctDNA monitoring during adjuvant therapy and follow-up: early warning of recurrence and guiding intervention

### 8.1 ctDNA molecular recurrence precedes clinical/imaging recurrence

Serial monitoring of ctDNA during adjuvant therapy and in routine follow-up after treatment completion can detect molecular-level recurrence, defined as the re-emergence or rising levels of ctDNA. This molecular detection consistently occurs months before clinical symptoms or radiographic evidence of relapse become apparent. Across a wide spectrum of solid tumors, studies have demonstrated that ctDNA-based detection of MRD or molecular recurrence provides a significant lead time over standard clinical and imaging-based surveillance. Reported lead times generally range from 2 to 12 months and vary with tumor type, tumor shedding biology, assay sensitivity, and sampling frequency. Representative examples include resected gastric cancer, in which ctDNA positivity preceded radiographic recurrence by a median of 6 months (146); early breast cancer, with median lead times of 11.7 months (147), up to 417 days (121), and approximately 1 year before distant metastasis in high-risk hormone receptor-positive disease (148); colorectal cancer liver metastases (3.1 months) (149); head and neck squamous cell carcinoma (108–253 days, and a median of 7.0 months in locally advanced disease) (150, 151); muscle-invasive bladder cancer treated with trimodality therapy (138 days) (152); and unresectable NSCLC treated with definitive radiotherapy (5.4 months) (153). Concordant observations in Merkel cell carcinoma (154), uveal melanoma (155), stage I and resected NSCLC (156, 157), gastroesophageal cancers (158), anal squamous cell carcinoma (159), urothelial carcinoma (160), and a pan-cancer tumor-informed analysis (5) establish ctDNA as a highly sensitive early-warning system for impending relapse (**Table 2**).

**Table 2:** Representative clinical studies of ctDNA across the neoadjuvant treatment continuum in solid tumors.

Study/cohort [ref]	Cancer type	Treatment setting	ctDNA assessment timing	Main clinical findings
I-SPY 2 (94, 172)	Breast (TNBC, HR+/HER2−)	Neoadjuvant chemotherapy ± investigational agents (adaptive platform)	Baseline, week 3, inter-regimen, pre-surgery	Week-3 clearance predicted pCR in TNBC; ctDNA positivity after NAC associated with markedly higher metastatic recurrence risk (HR 10.4)
Tumor-informed ultrasensitive assay cohort (95)	Breast (HER2+, TNBC)	Neoadjuvant therapy + surgery	Post-neoadjuvant, postoperative	Detectable ctDNA outperformed pCR status for prognostic stratification; ctDNA-negative patients had excellent 5-year iDFS
ABCSG-34 (173)	Breast	Neoadjuvant chemotherapy/endocrine therapy	Baseline, post-neoadjuvant	Baseline ctDNA positivity prognostic for inferior overall survival on long-term follow-up
TNBC mid-NAC cohort (63)	Breast (TNBC)	Neoadjuvant chemotherapy	Baseline, mid-treatment	ctDNA clearance by mid-NAC strongly associated with pCR
DYNAMIC (123, 124)	Colon (stage II)	Adjuvant decision after surgery	Postoperative (weeks 4–7)	Randomized: ctDNA-guided strategy reduced chemotherapy use without compromising recurrence-free survival — first randomized evidence of clinical utility
CIRCULATE-US (138); TRACC Part C (143)	Colon/CRC (stage II–III)	Adjuvant	Postoperative, serial	Ongoing randomized trials of MRD-guided treatment escalation and de-escalation
LARC prospective cohort (72)	Rectal (LARC)	Neoadjuvant chemoradiotherapy	Baseline, during and after nCRT	ctDNA combined with MRI TRG improved pCR prediction over MRI alone; post-nCRT driver-gene ctDNA associated with worse RFS
TNT/non-operative management series (77, 78)	Rectal (LARC)	Total neoadjuvant therapy	Post-TNT	ctDNA positivity has very high PPV for residual disease; negativity insufficient alone to select watch-and-wait
CRLM kinetics study (64)	CRC liver metastases	Neoadjuvant chemotherapy	After one cycle	≥50% decline in mean VAF predicted response and RFS, outperforming CEA/CA19-9
NADIM (186)	NSCLC (resectable stage IIIA)	Neoadjuvant nivolumab + chemotherapy	Baseline, post-neoadjuvant	Undetectable ctDNA after neoadjuvant therapy associated with improved PFS/OS, outperforming RECIST response
CTONG1804 (190); NEOTIDE/CTONG2104 (192)	NSCLC (incl. EGFR-mutant)	Neoadjuvant targeted therapy/immunochemotherapy	Serial perioperative	Post-neoadjuvant ctDNA negativity linked to pCR and superior survival; tumor-informed ctDNA identified non-responders
Camrelizumab + apatinib phase II (70)	NSCLC	Neoadjuvant immunotherapy + anti-angiogenic	Perioperative	ctDNA clearance significantly associated with improved event-free survival
Neoadjuvant ICI meta-analysis (67)	Pan-solid tumors	Neoadjuvant immune checkpoint inhibitors	Pre- and post-neoadjuvant	Lack of clearance predicted non-pCR (pooled sensitivity 0.98); confirmatory value of clearance limited by assay variability
Neoadjuvant ipilimumab + nivolumab (55)	Melanoma	Neoadjuvant immunotherapy	Baseline, post-treatment	ctDNA clearance after treatment correlated strongly with improved outcomes
ESCC nCRT cohort (99)	Esophageal (ESCC)	Neoadjuvant chemoradiotherapy + surgery ± adjuvant ICI	Post-nCRT, postoperative	MRD positivity predicted worse survival; adjuvant immunotherapy benefit confined to MRD-positive patients (hypothesis-generating)
MIBC cohorts (104, 105)	Bladder (MIBC)	Neoadjuvant chemotherapy + radical cystectomy	Pre- and post-neoadjuvant, postoperative	Pre-cystectomy ctDNA predicts residual tumor; postoperative MRD predicts relapse; prognostic value robust, treatment-directing utility investigational
Gastroesophageal neoadjuvant ICI cohort (75)	Gastroesophageal	Neoadjuvant immunotherapy	Post-induction	Failure to clear ctDNA associated with significantly worse RFS and OS

This capability to identify recurrence at a molecular stage, often termed “molecular residual disease” (MRD), fundamentally creates a novel therapeutic window. The stage where ctDNA is detectable but conventional imaging remains negative represents a unique opportunity for early intervention. This paradigm lays the essential groundwork for designing and conducting clinical trials that investigate preemptive or “molecular relapse”-directed therapies. The rationale is to initiate second-line or adjuvant therapies at this earlier, presumably lower-burden disease state, with the goal of eradicating MRD and preventing or delaying overt clinical recurrence. Evidence strongly supports this approach, as the presence of post-treatment ctDNA is a powerful prognostic marker associated with significantly worse disease-free and overall survival across cancers like gastric cancer (146), NSCLC (157), and breast cancer (66). Consequently, ongoing and future clinical trials are increasingly using ctDNA status to stratify patients at high risk of relapse and to guide treatment decisions. For example, trials are exploring whether initiating immunotherapy or targeted therapy upon detection of ctDNA MRD, rather than waiting for radiographic confirmation, can improve patient outcomes (161). This shift towards ctDNA-guided intervention studies promises to transform adjuvant therapy from a one-size-fits-all approach to a dynamic, risk-adapted strategy, potentially improving survival by targeting residual disease at its most vulnerable state. Nevertheless, a longer lead time is of clinical value only if effective early intervention exists: whether treating molecular relapse before radiographic confirmation improves survival is precisely the question that ongoing molecular relapse-directed trials must answer, and until they report, ctDNA surveillance after neoadjuvant therapy should not be regarded as standard of care.

### 8.2 ctDNA-guided post-recurrence treatment decisions

**Figure 7:**
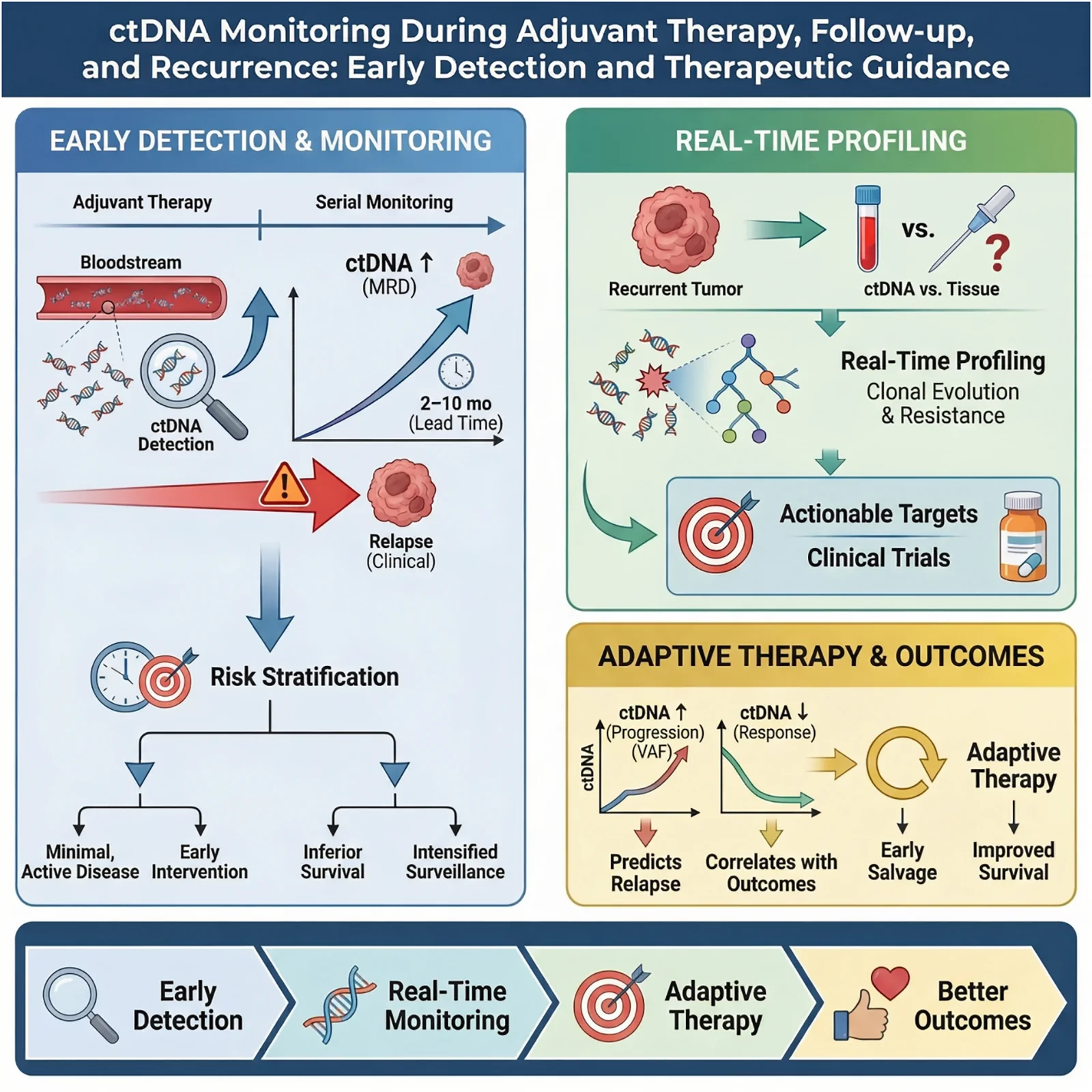
ctDNA surveillance during adjuvant therapy and follow-up. Molecular recurrence detected by ctDNA precedes clinical or imaging relapse, creating a potential window for early intervention and informing salvage treatment selection.

The detection of ctDNA upon disease recurrence offers a powerful, non-invasive method to reveal the molecular characteristics of the recurrent tumor, which may differ from the primary lesion. This capability is critical for guiding precise salvage therapy selection, potentially obviating the need for repeated invasive biopsies of metastatic sites. In recurrent or metastatic settings, ctDNA analysis can effectively capture the genomic evolution of the disease, identifying clinically actionable alterations that may not be present in archival primary tumor tissue. For instance, in patients with recurrent gastric or gastroesophageal junction adenocarcinoma, ctDNA profiling identified actionable genomic alterations in 36% of cases, with new alterations such as *ERBB2* amplifications and *PIK3CA* mutations emerging at recurrence that were not detected in the primary tumor in a significant proportion of patients (162). This demonstrates how ctDNA can reflect tumor clonal dynamics and heterogeneity, providing a real-time molecular snapshot to inform treatment. Similarly, in human papillomavirus-positive (HPV+) oropharyngeal squamous cell carcinoma (OPSCC), longitudinal monitoring of HPV16 ctDNA has been shown to correlate with treatment response and predict progression earlier than conventional imaging, with ctDNA responses observed an average of 70 days before radiographic changes (163). This early signal of treatment failure or success allows for timely therapeutic adjustments, such as switching to clinical trials or alternative regimens before clinical deterioration. The prognostic and predictive power of ctDNA extends across various malignancies. In breast cancer recurrence, the dynamics of ctDNA VAF are significantly associated with disease response, with increases in ctDNA often preceding clinical progression by a mean lead time of 6.2 months (164). In CRC, ctDNA detection after curative-intent treatment for metastatic disease is strongly associated with shorter recurrence-free and overall survival, serving as a robust biomarker for MRD and imminent relapse (165). This prognostic stratification is vital for identifying patients who may benefit from intensified surveillance or early intervention with salvage therapy. Furthermore, ctDNA analysis can guide specific therapeutic choices. Similar findings extend to less common settings, including recurrent glioblastoma monitored via tumor *in situ* fluid ctDNA (166) and uterine serous carcinoma or carcinosarcoma, in which persistently undetectable ctDNA after treatment is associated with prolonged survival (167). This enables clinicians to consider pre-emptive or consolidation therapies in high-risk patients. The utility of ctDNA also lies in its ability to monitor therapeutic efficacy dynamically. In ovarian cancer, an increase in ctDNA levels after the first cycle of chemotherapy was associated with an early treatment response and superior disease-free survival, positioning ctDNA as a potential early biomarker of chemosensitivity (168). For patients with resectable metastatic CRC, while preoperative ctDNA levels were not predictive, detectable postoperative ctDNA was strongly associated with recurrence, suggesting its value in post-operative risk assessment and guiding the potential need for additional systemic therapy (169). By providing a comprehensive, real-time view of the tumor’s molecular landscape at recurrence, ctDNA testing facilitates a more personalized approach to salvage therapy. It helps avoid the pitfalls of relying on historical tumor profiles that may no longer be representative, thereby optimizing drug selection, monitoring response, and ultimately aiming to improve outcomes for patients with recurrent solid tumors (**Figure 7**).

In appraising this surveillance literature, two caveats stand out. First, lead-time bias: earlier detection of relapse, however consistent, does not by itself improve outcomes, and the added survival value of ctDNA-triggered salvage therapy has yet to be demonstrated. Second, most post-recurrence genotyping data derive from metastatic cohorts, and their extrapolation to relapse after curative-intent neoadjuvant treatment—where re-biopsy is often feasible—requires confirmation. ctDNA-guided surveillance and intervention should therefore currently be offered within, rather than outside, clinical trials.

## 9 Application examples of ctDNA in neoadjuvant therapy for specific solid tumors

The preceding sections outlined the phase-specific principles of ctDNA use largely irrespective of histology. To avoid repetition, this section consolidates the evidence for the three tumor types in which it is most mature—breast cancer, colorectal cancer, and NSCLC—as representative examples; representative studies across these and other tumor types are summarized in **Table 2**.

### 9.1 Breast cancer

In HER2-positive breast cancer, the dynamic changes in ctDNA serve as a powerful predictive biomarker for pCR rates following neoadjuvant therapy, particularly anti-HER2 targeted treatments. Studies have consistently demonstrated that the clearance of ctDNA during neoadjuvant chemotherapy (NAC) is strongly associated with achieving pCR and improved long-term outcomes. For instance, research from the I-SPY 2 TRIAL showed that patients who remained ctDNA-positive three weeks after initiating paclitaxel-based NAC were significantly more likely to have residual disease (83% non-pCR) compared to those who cleared ctDNA (52% non-pCR), with an odds ratio of 4.33 (94). Furthermore, in patients who did not achieve pCR, the presence of ctDNA post-NAC was a potent indicator of a significantly increased risk of metastatic recurrence, with a hazard ratio of 10.4 (94). This underscores the prognostic value of ctDNA dynamics beyond the traditional pCR endpoint. Specifically for HER2-positive disease, investigations reveal that ctDNA positivity after neoadjuvant therapy is associated with inferior recurrence-free survival, even among patients who achieve pCR, suggesting that ctDNA detection post-treatment identifies a subset of patients with minimal residual disease who may benefit from escalated adjuvant therapy, such as trastuzumab emtansine (T-DM1) (47). The ability of ctDNA to reflect real-time tumor burden and treatment efficacy makes it a critical tool for personalizing neoadjuvant strategies in HER2-positive breast cancer, potentially allowing for early therapeutic adjustments. In contrast, for hormone receptor-positive/HER2-negative (HR+/HER2-) breast cancer, ctDNA analysis plays a pivotal role in monitoring the emergence of endocrine therapy resistance, particularly through the detection of *ESR1* mutations. While the provided references focus on broader ctDNA dynamics in HR+/HER2- disease, the principle is well-established: ctDNA profiling can identify actionable resistance mutations that guide subsequent treatment decisions, such as switching from aromatase inhibitors to selective estrogen receptor degraders or combining endocrine therapy with CDK4/6 inhibitors (83). For example, the DANCER trial investigated ctDNA-guided neoadjuvant therapy with the CDK4/6 inhibitor dalpiciclib plus aromatase inhibitors in HER2-negative luminal B breast cancer, identifying ctDNA clearance as a candidate predictive biomarker (170). More broadly, in HR+/HER2- breast cancer receiving neoadjuvant endocrine therapy, pre-treatment ctDNA detection is associated with higher pathological stage and residual cancer burden, while persistent ctDNA detection pre-surgery correlates with a higher risk of distant recurrence, highlighting its prognostic utility (171). Thus, across major breast cancer subtypes, ctDNA provides a non-invasive means to predict therapeutic response and monitor clonal evolution, directly informing precision treatment strategies in the neoadjuvant setting.

**Figure 8:**
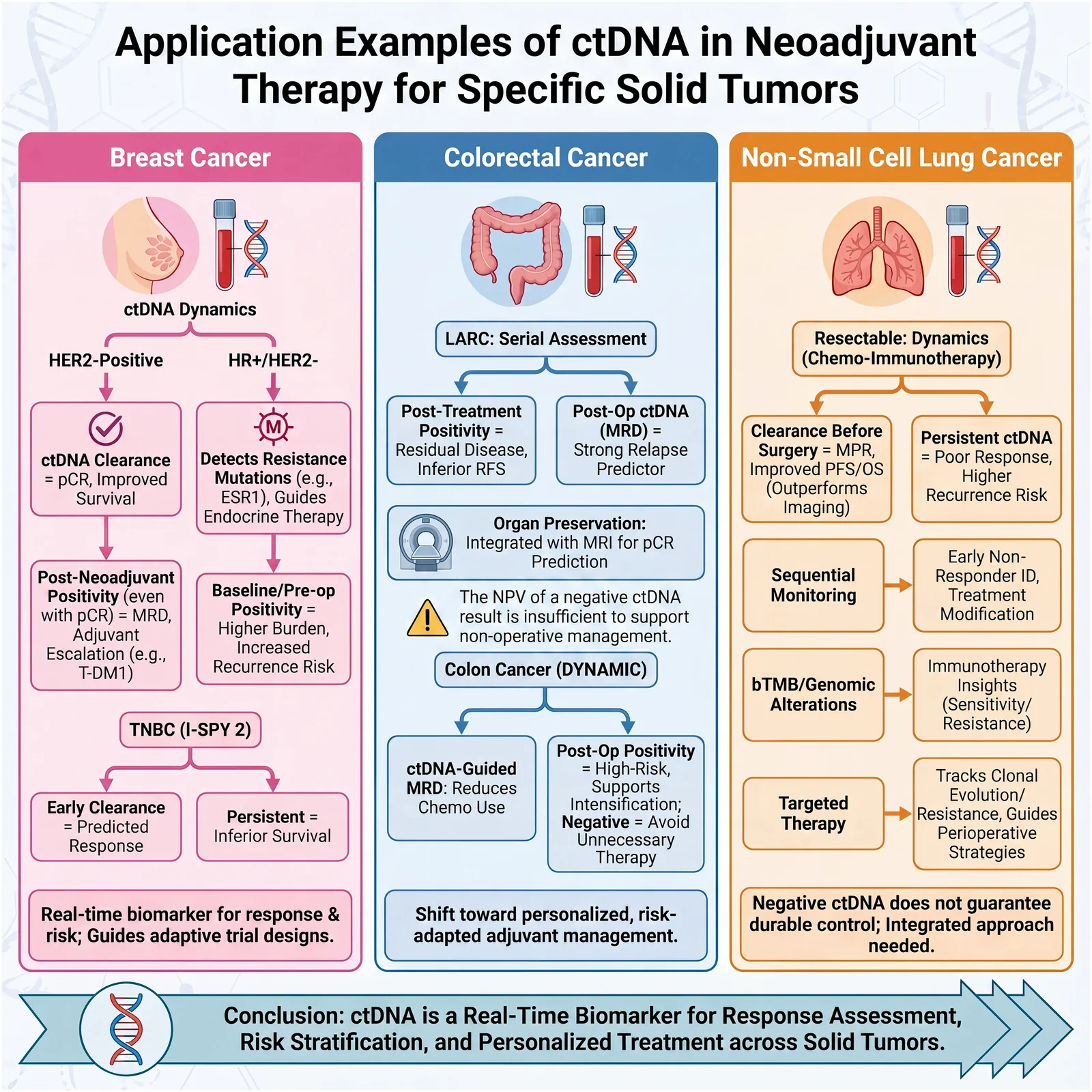
Representative clinical applications of ctDNA in breast cancer, colorectal cancer, and non-small cell lung cancer undergoing neoadjuvant therapy.

The integration of ctDNA analysis into adaptive platform clinical trials, such as the phase II I-SPY 2 trial, represents a transformative approach to neoadjuvant therapy personalization and real-time treatment arm allocation. The I-SPY 2 trial is a groundbreaking adaptive design that uses biomarker signatures, including ctDNA, to assign patients with high-risk early-stage HER2-negative breast cancer to different investigational therapies alongside standard NAC (172). Within this framework, serial personalized ctDNA analysis has been employed to investigate subtype-specific differences in ctDNA shedding and its clinical significance. Research from I-SPY 2 demonstrated that ctDNA positivity rates before, during, and after NAC are higher in TNBC than in HR+/HER2- breast cancer (172). Importantly, early clearance of ctDNA just three weeks after treatment initiation was found to predict a favorable response to NAC specifically in TNBC, but not in the HR+/HER2- subtype (172). This finding is crucial for adaptive trial design, as it suggests that ctDNA dynamics can serve as an early intermediate endpoint to identify responders and non-responders rapidly, allowing for potential therapy redirection within the trial protocol. The trial data further established that ctDNA positivity at any point is associated with reduced distant recurrence-free survival in both TNBC and HR+/HER2- subtypes, while ctDNA negativity after NAC correlates with improved outcomes, even in patients with extensive residual cancer (172). Based on these robust findings, the I-SPY 2 trial has prospectively incorporated ctDNA testing to evaluate its utility in redirecting therapy to improve response and prognosis, moving from retrospective biomarker analysis to proactive clinical utility (172). This model exemplifies how ctDNA can be seamlessly integrated into complex trial architectures to enable real-time, biomarker-driven treatment assignments. Beyond I-SPY 2, other trials are also exploring this paradigm. For instance, the Pathologic Response Evaluation and Detection in Circulating Tumor-DNA study prospectively evaluated an ultrasensitive, tumor-informed ctDNA assay in HER2-positive and TNBC patients, finding that detectable ctDNA after neoadjuvant therapy provided markedly superior prognostic stratification compared to pCR status alone, identifying patients at extreme risk of recurrence who are candidates for therapeutic escalation (95). Similarly, the phase II ABCSG-34 trial incorporated tumor-informed ctDNA assays to assess dynamics during neoadjuvant therapy, with long-term follow-up confirming baseline ctDNA positivity as a prognostic marker for inferior overall survival (173). These trials collectively validate the role of ctDNA as a dynamic biomarker within adaptive platforms, facilitating the shift from a one-size-fits-all neoadjuvant approach to a more nimble, response-adapted strategy that aims to improve outcomes by matching therapy to real-time molecular evidence of disease burden and treatment efficacy (**Figure 8**).

### 9.2 Colorectal cancer

In LARC, the status of ctDNA before and after nCRT has emerged as a pivotal factor for predicting pCR, recurrence-free survival, and guiding subsequent management strategies such as the “watch and wait” (W&W) approach or adjuvant chemotherapy. A systematic review and meta-analysis of observational studies found that ctDNA detection at any timepoint in patients undergoing neoadjuvant treatment for non-metastatic cancer, including rectal cancer, was strongly associated with recurrence, with the association being particularly robust at post-treatment and post-surgery timepoints (174). Specifically, ctDNA positivity after nCRT completion (post-neoadjuvant therapy) is a powerful prognostic indicator. A prospective cohort study in LARC patients demonstrated that the detection of potential colorectal cancer driver genes in ctDNA after nCRT was significantly associated with worse recurrence-free survival (RFS) (72). Furthermore, ctDNA status after surgery, representing MRD, is a critical determinant of recurrence risk. Studies consistently show that patients with detectable ctDNA (MRD-positive) after curative-intent surgery have a significantly higher risk of recurrence and worse survival compared to those who are ctDNA-negative (175, 176). This prognostic value holds true even in patients undergoing upfront surgery without neoadjuvant therapy, where postoperative ctDNA positivity was a robust biomarker predicting recurrence risk and benefit from adjuvant chemotherapy (177). In the context of organ preservation, ctDNA analysis is being investigated to complement clinical assessments for selecting patients for a W&W strategy after achieving a cCR. As discussed in Section 5.2, ctDNA positivity after TNT has a very high positive predictive value for residual disease, whereas a negative test cannot exclude microscopic disease; ctDNA should therefore complement, not replace, clinical and radiological evaluation when selecting patients for non-operative management (77, 78). The integration of ctDNA dynamics with magnetic resonance imaging (MRI) findings, such as tumor regression grade (mrTRG), has been shown to improve the predictive performance for pCR compared to using either modality alone, potentially refining patient selection for treatment de-escalation or intensification (72). Thus, serial ctDNA assessment provides a real-time, molecular snapshot of tumor burden, offering a critical tool for risk stratification and personalized decision-making in the multidisciplinary management of LARC.

Concurrently, ctDNA-based MRD detection is fundamentally reshaping the decision-making paradigm for adjuvant chemotherapy in stage II colon cancer. The traditional approach to adjuvant therapy in stage II disease is challenged by the fact that only a subset of patients benefits, while many are exposed to unnecessary toxicity. ctDNA analysis post-surgery addresses this by identifying patients with molecular evidence of residual disease who are at high risk for recurrence and are most likely to benefit from adjuvant treatment. Landmark prospective studies and randomized trials have established the clinical utility of this approach. The DYNAMIC trial, highlighted in reviews of practice-changing studies, demonstrated that a ctDNA-guided strategy for adjuvant therapy in stage II colon cancer could safely reduce chemotherapy exposure without compromising long-term outcomes, effectively de-escalating treatment for ctDNA-negative patients (123, 124). This is supported by a large body of evidence showing that postoperative ctDNA positivity is among the strongest known biomarkers for recurrence risk in resected colorectal cancer, with clear separation in DFS and OS between MRD-positive and MRD-negative patients (124, 178). A meta-analysis confirmed that ctDNA detection after surgery was significantly associated with shorter RFS, and this association was even stronger after patients completed adjuvant chemotherapy, underscoring ctDNA’s role in signifying chemotherapy-resistant disease (179). Tumor-informed assays, which track patient-specific mutations, have shown high sensitivity and specificity for MRD detection, providing a highly personalized risk assessment (180, 181). The prognostic power of ctDNA often surpasses that of traditional clinicopathological risk factors and serum carcinoembryonic antigen (CEA) levels (182). Consequently, clinical guidelines are beginning to incorporate these findings. For instance, the updated Chinese Anti-Cancer Association (CACA) guidelines have supplemented the role of ctDNA in treatment decision-making, reflecting its growing integration into standard care (183). This paradigm shift enables a more precise allocation of adjuvant therapy: intensification for ctDNA-positive patients at high risk and potential omission for ctDNA-negative patients at low risk, moving beyond a one-size-fits-all guideline-based approach towards truly personalized, precision oncology in the adjuvant setting for colon cancer (184, 185) (**Figure 8**).

### 9.3 Non-small cell lung cancer

In the context of neoadjuvant immunotherapy for resectable NSCLC, the clearance of ctDNA has emerged as a significant biomarker strongly correlated with MPR and serves as a tool for the early identification of non-responders. Data from pivotal clinical trials underscore this relationship. For instance, in the NADIM phase II trial, undetectable ctDNA levels after neoadjuvant treatment with nivolumab plus chemotherapy were significantly associated with improved progression-free survival (PFS) and OS, with ctDNA analysis outperforming radiologic RECIST criteria in predicting survival outcomes (186). Similarly, a study evaluating ctDNA dynamics during neoadjuvant treatment (including immunotherapy combined with chemotherapy, dual immunotherapy, or chemotherapy alone) found that ctDNA clearance was highly concordant with pathologic response, demonstrating 100% sensitivity and 83.33% specificity for an overall accuracy of 91.67% (187). This study also noted that detectable ctDNA after neoadjuvant therapy (pre-surgery) trended towards inferior RFS (187). The association between ctDNA clearance and superior pathological outcomes is further supported by data from trials of neoadjuvant chemo-immunotherapy, where ctDNA clearance before surgery was linked to higher rates of MPR (69). This predictive capacity allows for the early identification of patients deriving suboptimal benefit from neoadjuvant immunotherapy, enabling potential therapeutic adjustments (188). The utility of ctDNA extends beyond a simple binary measure; its sequential testing can serve as a secondary indicator to guide the modification of treatment programs (189). Perioperative ctDNA clearance was likewise associated with improved event-free survival in the camrelizumab plus apatinib trial noted earlier (70). Furthermore, post-neoadjuvant ctDNA negativity has been linked to a higher likelihood of achieving pCR and superior survival benefits, as observed in the CTONG1804 trial (190). The prognostic power of post-treatment ctDNA status is robust, with positive testing strongly correlated with a lower MPR or pCR rate (189). Collectively, these findings validate ctDNA clearance as a dynamic, non-invasive surrogate for pathological response and an early indicator of treatment efficacy in the neoadjuvant setting for operable NSCLC.

Beyond simple clearance, the dynamic changes in blood-based tumor mutational burden (bTMB) derived from ctDNA are also intricately linked to immunotherapy response in NSCLC. While the predictive value of baseline tissue TMB or PD-L1 expression has shown limitations in neoadjuvant studies (186), the evolution of bTMB during treatment offers a more nuanced view of tumor-immune interactions. Although not all studies have found baseline TMB predictive of survival in trials like NADIM (186), the dynamic profiling of ctDNA, including mutational landscape analysis, provides critical insights into treatment efficacy and resistance mechanisms. The ctDNA analysis enables real-time monitoring of tumor genomic alterations and clonal evolution, which is particularly valuable for assessing response to neoadjuvant targeted therapies and immunotherapies (191). For instance, in patients with driver mutations such as EGFR, monitoring ctDNA during neoadjuvant therapy can help identify those non-responsive to immunochemotherapy, as demonstrated in the NEOTIDE/CTONG2104 trial of neoadjuvant sintilimab plus chemotherapy in EGFR-mutant NSCLC (192). This study utilized tumor-informed ctDNA detection to help identify non-responders (192). The ability to track specific mutations, such as HER2 and TP53 in a case of perioperative trastuzumab deruxtecan treatment, allows for the detection of molecular progression and guides timely treatment adjustments (193). Furthermore, longitudinal liquid biopsy analyses in advanced NSCLC reveal that dynamic subclonal evolution drives early treatment resistance, a principle that likely extends to the neoadjuvant setting (194). The assessment of ctDNA status, including variant allele frequencies and the presence of specific resistance mutations, can predict the efficacy of both neoadjuvant and adjuvant therapies (191). For example, in the context of neoadjuvant targeted therapy for MET exon 14 skipping mutations, ctDNA clearance following treatment was associated with improved clinical outcomes (194). However, it is crucial to note that a negative postoperative ctDNA finding may not always predict safe discontinuation of adjuvant targeted therapy, as evidenced by a case report where rapid metastasis occurred despite negative ctDNA after neoadjuvant crizotinib (195). This highlights the complexity of using ctDNA dynamics alone and underscores the need for integrated biomarker approaches. The evolving role of ctDNA analysis, including bTMB dynamics, is thus central to refining patient selection, monitoring therapeutic response, and understanding mechanisms of resistance in the era of perioperative chemo-immunotherapy and targeted therapy for resectable NSCLC (69) (**Figure 8**).

Viewed across tumor types (**Table 2**), the same principles recur—baseline prognostication, kinetic response prediction, and MRD-based risk stratification—but the maturity of the evidence differs markedly: colorectal cancer is currently the only setting with completed randomized evidence of clinical utility, whereas the breast and lung cancer data, although extensive, remain observational or embedded in single-arm and platform trials. Differences in shedding biology, preferred assay strategies, and endpoints caution against uncritical extrapolation of thresholds or algorithms from one tumor type to another.

## 10 Current challenges, limitations, and future prospects

### 10.1 Urgent need for technical standardization and clinical validation

The widespread clinical adoption of ctDNA analysis is critically hampered by a lack of standardization across the entire analytical workflow, from pre-analytical sample handling to final reporting, which impedes cross-center comparability and robust clinical implementation (196). Significant variability exists in pre-analytical factors such as blood collection tube types, plasma processing protocols, and DNA extraction methods, all of which can profoundly affect the quantity and quality of the isolated cell-free DNA, leading to inconsistent results (197, 198). For instance, different commercial cfDNA isolation kits demonstrate varying recovery rates for short DNA fragments typical of ctDNA, directly impacting assay sensitivity (26). Furthermore, the analytical phase suffers from a lack of harmonization in assay design, sequencing depth, and bioinformatic pipelines for variant calling, with numerous commercially available tests employing different gene panels and technologies (199, 200). This methodological heterogeneity is compounded by the absence of universally accepted, clinically validated thresholds for defining critical states such as ctDNA “clearance” or “positivity” for MRD, making it difficult to translate research findings into standardized clinical decisions (201, 202). For example, while studies show that postoperative ctDNA positivity is a strong prognostic marker in colorectal cancer, the specific variant allele frequency that constitutes a positive result is not standardized, hindering its use as a definitive guide for adjuvant therapy (124). Reporting formats also vary widely, lacking consistency in how mutations, variant allele frequencies, and tumor fractions are communicated to clinicians, further complicating integration into routine workflows (199). To overcome these barriers, there is an urgent need for the development and widespread adoption of universal standard operating procedures (SOPs) and reporting frameworks, such as those proposed by initiatives like MIBlood-EV and the updated MISEV guidelines, which aim to enhance rigor and reproducibility by standardizing the reporting of pre-analytical variables and quality control metrics (203, 204). Collaborative efforts by international consortia and professional societies are essential to establish these minimum performance standards for assays, define clinically relevant cut-off values, and create harmonized reporting templates (205, 206).

**Figure 9:**
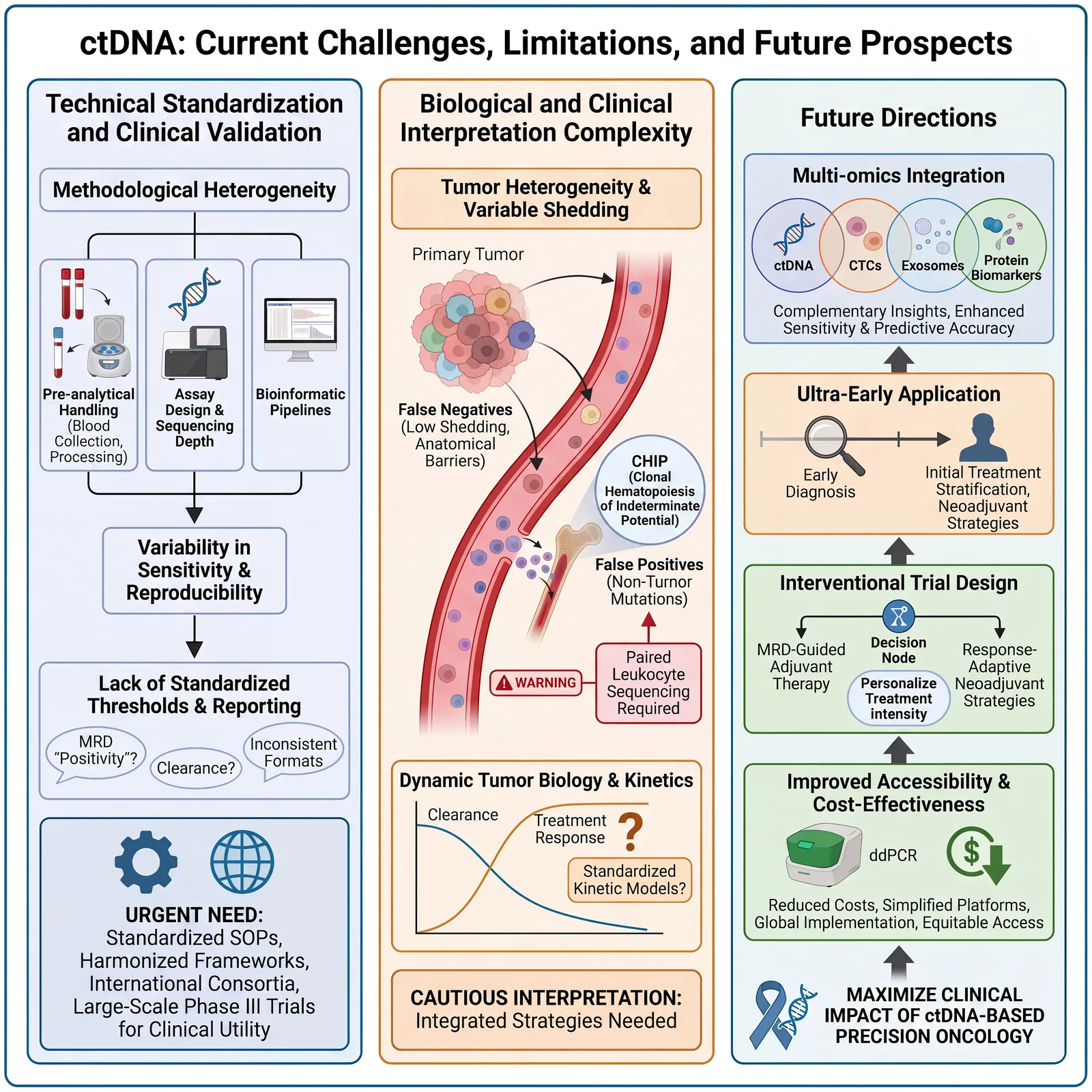
Current challenges and future directions for the clinical implementation of ctDNA. Key limitations include lack of standardization and interpretive complexity; future priorities include multi-omics integration, interventional trial designs, earlier application, and improved cost-effectiveness and accessibility.

Beyond technical harmonization, the definitive integration of ctDNA into standard clinical practice hinges on demonstrating its clinical utility through large-scale, prospective, multi-center Phase III trials (207). While a robust body of evidence supports the clinical validity of ctDNA—showing strong prognostic associations with recurrence and survival across various cancers like breast, colorectal, and lung cancer—proof that acting on ctDNA results to modify therapy ultimately improves patient outcomes, particularly OS, remains the critical unmet need (124, 208, 209). For instance, in early-stage breast cancer, ctDNA detection after neoadjuvant therapy is consistently linked to worse relapse-free survival, but interventional trials are required to determine if escalating adjuvant treatment based on a positive ctDNA result can alter this natural history (210, 211). Similarly, in colorectal cancer, although the DYNAMIC trial demonstrated that a ctDNA-guided strategy could reduce adjuvant chemotherapy use without compromising outcomes, further trials are needed to confirm that ctDNA-directed escalation therapy improves survival in high-risk patients (124). The clinical utility of ctDNA for dynamic monitoring in advanced disease also requires rigorous validation; while early changes in ctDNA levels correlate with response to immune checkpoint inhibitors in non-small cell lung cancer, it must be proven that switching therapies based on rising ctDNA before radiographic progression improves patient outcomes (212, 213). These trials must employ standardized, analytically validated assays with predefined thresholds and clear protocols for clinical action to generate conclusive evidence (205, 206). Until such high-level evidence is available, recommendations from bodies like the ESMO Precision Medicine Working Group caution against using ctDNA for MRD detection to guide routine clinical treatment decisions outside of clinical trials, emphasizing that tissue-based testing remains preferred in many scenarios due to current limitations in detecting fusions and copy number alterations via liquid biopsy (206). Therefore, the concerted pursuit of both technical standardization and rigorous clinical validation in prospective interventional studies is the indispensable pathway to transforming ctDNA from a promising research tool into a cornerstone of precision oncology practice (**Figure 9**). Recent tumor-specific appraisals reach the same conclusion; in muscle-invasive bladder cancer, for example, ctDNA currently offers robust prognostic information, whereas its role in directing perioperative and systemic therapy still awaits prospective validation (105).

### 10.2 Complexity of biological and clinical interpretation

The biological and clinical interpretation of ctDNA in the context of neoadjuvant therapy for solid tumors is fraught with complexities that can significantly impact its reliability as a biomarker. A primary challenge is tumor heterogeneity, which can lead to false-negative ctDNA results. This occurs because certain metastatic foci or residual disease sites may not release, or release only minimal amounts of, DNA into the bloodstream. This spatial heterogeneity means that a ctDNA assay may fail to detect the presence of disease even when viable tumor cells persist, particularly in tumors with low proliferative rates or those that are anatomically sequestered, such as brain tumors where ctDNA detection in plasma is notably lower compared to other sites (214). For instance, in non-muscle-invasive bladder cancer, the concordance of somatic variants between tumor DNA and plasma ctDNA was significantly higher in patients with larger tumor size and higher stage, indicating that tumor burden and characteristics directly influence ctDNA shedding (215). Furthermore, modeling studies suggest that spatially variable rates of cell death, such as elevated apoptosis on the tumor edge induced by therapy, can bias the clonal composition detected in ctDNA, potentially over-representing invasive clones and under-representing others, thereby distorting the true tumor genomic landscape (216). This biological limitation underscores that a negative ctDNA test does not unequivocally rule out the presence of MRD, necessitating cautious interpretation, especially in a post-neoadjuvant setting where the goal is to identify patients at highest risk of relapse.

The kinetics of ctDNA clearance present another layer of interpretive complexity, as they vary considerably based on tumor type, specific treatment modality, and individual patient factors. The dynamic changes in ctDNA levels, or molecular response, require sophisticated mathematical models for accurate interpretation to correlate with long-term clinical outcomes such as progression-free and overall survival (217). For example, in patients with various solid tumors treated with immunotherapy, a decline in ctDNA levels as early as 3–4 weeks after treatment initiation has been correlated with improved survival, while persistence or increase is associated with poor outcomes (68, 218). However, the rate and completeness of clearance are not uniform. In early-stage breast cancer after neoadjuvant chemotherapy, the presence of ctDNA post-treatment (pre-surgery) was strongly associated with disease relapse, whereas its absence was reassuring, but the sensitivity of detection at these time points can be low (219). The half-life of ctDNA is short, allowing for rapid assessment of treatment effect, but this also means that the timing of blood draws is critical. Kinetic models must account for factors like the initial tumor burden, the mechanism of action of the therapy (e.g., cytotoxic chemotherapy vs. immunotherapy), and the patient’s clearance physiology. Without standardized kinetic models and validated thresholds for what constitutes a significant molecular response, comparing results across studies and applying them in clinical practice remains challenging (93).

A persistent and major source of false-positive results in ctDNA analysis is the presence of CHIP variants. These are somatic mutations originating from hematopoietic stem cells that are acquired with age and can be detected in cell-free DNA, mimicking tumor-derived mutations (220). Distinguishing true tumor-derived variants from CHIP is paramount for accurate genotyping to guide therapy, such as avoiding the erroneous targeting of a CHIP variant with a targeted drug. The established method to mitigate this is through paired sequencing of leukocyte-derived DNA (germline control) from the same blood sample or a separate buffy coat specimen (221). This allows for the subtraction of mutations found in the white blood cells, which are likely of hematopoietic origin, from those found only in the plasma cfDNA, which are more likely to be tumor-derived. Failure to perform this essential step can lead to misinterpretation of a patient’s mutation profile, potentially excluding them from beneficial therapies or incorrectly assigning them to treatments with no expected efficacy. This requirement adds a layer of analytical and logistical complexity to ctDNA testing workflows but is non-negotiable for ensuring specificity, particularly when using broad, tumor-agnostic sequencing panels that are not informed by a patient’s specific tumor genotype (222) (**Figure 9**).

### 10.3 Future directions

- Multi-omics Integration: Combining circulating tumor cells (CTCs), exosomes, protein markers, etc., to construct a more comprehensive liquid biopsy profile.

The future of ctDNA application in the neoadjuvant setting hinges on its integration within a broader multi-omics liquid biopsy framework to overcome inherent limitations and construct a more holistic, real-time molecular portrait of the tumor. While ctDNA excels at capturing tumor-specific genomic alterations and burden, its sensitivity can be compromised by low-shedding tumors or biological confounders like clonal hematopoiesis (1). Combining ctDNA with other circulating analytes, such as circulating tumor cells (CTCs), exosomes, and protein markers, can provide complementary information on tumor biology, heterogeneity, and therapeutic response. For instance, CTCs offer insights into metastatic potential and viable tumor cell dissemination, and their presence has been correlated with poor outcomes in cancers like PDAC even in patients achieving a pCR (223). Exosomes, secreted by viable cells, emerge earlier in disease progression and preserve multi-omic cargo (including DNA, RNA, and proteins) that mirrors intratumor heterogeneity, potentially offering a more sensitive tool for early detection and monitoring than ctDNA or CTCs alone (224). Furthermore, integrating protein biomarkers, such as CEA or cancer antigen 19-9 (CA19-9), with ctDNA analysis has been shown to improve the overall assessment of treatment response and recurrence prediction. In locally advanced gastric cancer, the combination of ctDNA and tumor markers (TMs) increased the positive detection rate to 94.9% and provided better agreement with pathological response than either marker alone (225). This multi-analyte approach is also being explored in prospective trials. For example, the PRIMO study in LARC combines serial multiparametric MRI with analyses of CTCs, ctDNA, and tumor-infiltrating lymphocytes to develop a non-invasive response prediction model (226). Similarly, in TNBC, combining metabolomics, exosomal miRNAs, and flow cytometry for myeloid-derived suppressor cells (MDSCs) has generated predictive models for neoadjuvant chemotherapy response (227). The convergence of these diverse data streams—genomic, transcriptomic, proteomic, and cellular—within an integrated analytical platform, potentially powered by artificial intelligence, promises to refine risk stratification, enable earlier detection of MRD, and guide truly personalized, response-adapted treatment strategies by providing a more complete and dynamic understanding of tumor dynamics than any single biomarker can offer alone (1).

- Ultra-early Application: Exploring applications in the screening and diagnosis stages, for earlier linkage with neoadjuvant therapy.

A transformative future direction involves pushing the application of ctDNA analysis earlier in the cancer care continuum, from the metastatic and adjuvant settings into screening, diagnosis, and the very initial stages of neoadjuvant therapy planning. The goal is to enable ultra-early intervention by identifying high-risk individuals and characterizing tumors non-invasively before or at the point of diagnosis, thereby facilitating a more seamless and timely initiation of neoadjuvant strategies. Evidence suggests that ctDNA is detectable at diagnosis in a significant proportion of patients with localized cancers and carries prognostic significance. In early breast cancer, baseline ctDNA detection rates range from 38% to over 77%, and its presence is associated with higher tumor burden, more aggressive features (like high Ki67), and independently predicts worse recurrence-free and overall survival (66, 228, 229). Similarly, in localized PDAC, pre-treatment ctDNA detection, particularly KRAS mutations, is associated with more advanced disease and independently predicts worse progression-free survival in patients undergoing neoadjuvant chemotherapy (230). This prognostic value at diagnosis positions ctDNA as a potential tool for triaging patients towards more intensive neoadjuvant regimens or clinical trials. For instance, detecting specific driver mutations like KRASG12C in the plasma of patients with resectable lung adenocarcinoma could identify candidates for neoadjuvant trials with targeted KRAS inhibitors (231). Furthermore, ctDNA shows promise in improving patient selection for neoadjuvant therapy itself. In localized proficient mismatch repair (pMMR) colon cancer, pre-operative ctDNA positivity is strongly associated with higher pathological stage and a significantly increased risk of early recurrence, suggesting its potential to identify the high-risk subgroup that might benefit most from neoadjuvant chemotherapy, although its additive value to clinical models requires further validation (232). The concept extends to using ctDNA for ultra-early response assessment. Studies in colorectal liver metastases and breast cancer demonstrate that ctDNA dynamics as early as after one cycle of neoadjuvant therapy can predict pathological and radiological response, outperforming traditional markers like CEA (64, 233). This capability to provide a real-time, molecular readout of treatment efficacy during the traditionally opaque “black box” period of neoadjuvant therapy could revolutionize care by allowing for rapid therapy adaptation—intensifying treatment for non-responders or de-escalating for those with rapid molecular clearance—long before surgical pathology is available (1). Realizing this ultra-early application paradigm requires overcoming challenges related to the sensitivity of detecting very low tumor fractions in screening scenarios and the standardization of assays, but it represents a critical step towards intercepting cancer at its most treatable stage.

- Interventional Clinical Trial Design: Promoting more “interventional” clinical trials with ctDNA as a decision node, such as MRD-guided adjuvant therapy studies, to confirm its clinical utility.

The definitive translation of ctDNA from a prognostic biomarker to a tool that guides therapeutic decisions requires a paradigm shift towards prospective, interventional clinical trials where ctDNA status directly informs treatment allocation. The current evidence is largely observational, robustly establishing that detectable ctDNA after neoadjuvant therapy or surgery (molecular residual disease, MRD) is a powerful predictor of recurrence across multiple solid tumors, including breast, colorectal, lung, and pancreatic cancers (122, 234–236). However, to demonstrate clinical utility, trials must test whether acting upon this information improves patient outcomes. The most active area of such research is in MRD-guided adjuvant therapy. The core hypothesis is that patients with detectable ctDNA after curative-intent treatment harbor MRD and are at high risk of recurrence; therefore, they may benefit from additional, escalated adjuvant therapy (e.g., chemotherapy, immunotherapy, or targeted therapy). Conversely, patients who are ctDNA-negative post-treatment likely have a very low risk of recurrence and could be spared the toxicity of unnecessary adjuvant treatment. This approach is being actively investigated. For example, in the TRICIA trial for TNBC patients with residual disease after neoadjuvant chemotherapy, ctDNA monitoring was used to stratify risk, and clearance of ctDNA during adjuvant capecitabine was associated with a good prognosis (237). In colorectal cancer, multiple ongoing trials are evaluating whether ctDNA-positive patients after surgery should receive adjuvant chemotherapy or novel agents (238). Similarly, in NSCLC, post-operative ctDNA positivity is being explored as a criterion for adjuvant therapy selection (239). Beyond adjuvant decisions, ctDNA dynamics during neoadjuvant therapy itself are a compelling decision node for interventional trials. Rapid ctDNA clearance could signal high sensitivity, prompting studies on treatment de-escalation (e.g., shorter duration or omission of certain agents), while persistent ctDNA could trigger a switch to an alternative, more intensive regimen before surgery (1). Trials like the NOMINATE study in LARC are already incorporating ctDNA analysis to understand its correlation with response to total neoadjuvant therapy and non-operative management decisions (240). Furthermore, ctDNA can guide the selection of targeted therapies in the neoadjuvant setting based on the genomic profile identified in plasma, as suggested for MET or KRAS-altered cancers (195, 231). The success of these interventional trials depends on using highly sensitive and standardized ctDNA assays, clearly defined ctDNA-positive/negative thresholds, and appropriate clinical endpoints. Their results are eagerly awaited, as they hold the potential to establish ctDNA as a cornerstone of precision oncology, transforming treatment paradigms by moving from a one-size-fits-all approach to a dynamic, biomarker-driven strategy that personalizes therapy based on real-time molecular evidence of disease burden and response.

- Cost-effectiveness and Accessibility: Driving down technological costs and exploring its application models in different healthcare resource environments.

For the transformative potential of ctDNA monitoring to be realized globally, addressing the critical challenges of cost-effectiveness and equitable accessibility is paramount. Currently, the high cost of NGS-based ctDNA assays, especially personalized tumor-informed approaches, poses a significant barrier to widespread clinical adoption, particularly in resource-limited settings (189). The economic burden includes not only the test itself but also the infrastructure for bioinformatics analysis and the expertise required for interpretation. Therefore, future efforts must focus on driving down technological costs through innovation, economies of scale, and the development of more affordable yet reliable platforms. Promising avenues include refining ddPCR for high-sensitivity detection of a limited set of patient-specific mutations, which can be more cost-effective for longitudinal MRD monitoring than broad NGS panels (59, 241). Additionally, the exploration of tumor-agnostic methods, such as methylation-based sequencing or fragmentomics analysis, which do not require prior tumor tissue sequencing, could streamline workflows and reduce costs (122, 242). For instance, a universal methylation-specific ddPCR assay for sarcomas has been developed to detect ctDNA across various histotypes without needing tumor tissue, demonstrating potential for broader application (20). Beyond cost reduction, developing and validating application models tailored to different healthcare resource environments is essential. In high-resource settings, ctDNA may be integrated into comprehensive multimodal monitoring programs alongside imaging. In low- and middle-income countries, a more pragmatic approach might involve using ctDNA at key, sparse decision points, such as post-neoadjuvant therapy assessment to guide the need for surgery or post-operatively to identify the subset of patients who truly require adjuvant chemotherapy, thereby optimizing the use of limited resources. Studies must formally evaluate the cost-effectiveness of ctDNA-guided strategies. For example, avoiding adjuvant chemotherapy in ctDNA-negative patients could save substantial costs and prevent toxicity, while targeting intensified therapy only to ctDNA-positive high-risk patients could improve outcomes in a cost-efficient manner (122). Furthermore, exploring alternative biofluids like urine for ctDNA detection offers a less invasive and potentially more accessible option, though current sensitivity is lower than plasma (243, 244). Collaborative international consortia and public-private partnerships will be crucial to standardize assays, validate their utility across diverse populations, and create sustainable pricing models. Ultimately, the goal is to ensure that the precision oncology benefits enabled by ctDNA are not confined to well-funded institutions but become a viable tool for improving cancer care and outcomes worldwide, regardless of geographic or economic boundaries (**Figure 9**).

## 11 Conclusion

The integration of ctDNA into the management of neoadjuvant therapy for solid tumors represents a paradigm shift in precision oncology. From baseline risk assessment to early response prediction, dynamic monitoring during treatment, postoperative MRD evaluation, and recurrence surveillance, ctDNA has emerged as a powerful complement to conventional imaging and histopathological methods—albeit one whose role in directing treatment, in most settings, is still being defined in prospective trials. Its ability to provide real-time, tumor-specific molecular insights allows for a more nuanced understanding of tumor biology and treatment dynamics, fundamentally altering how clinicians approach neoadjuvant strategies.

From an expert perspective, the evolution of ctDNA in this setting underscores a critical transition from static, snapshot assessments to a continuous, fluid monitoring paradigm. The dynamic changes in ctDNA levels offer a window into therapeutic efficacy that is both earlier and more sensitive than traditional radiological or pathological evaluations. This capability is particularly transformative in the context of MRD detection, where ctDNA enables the re-stratification of recurrence risk with unprecedented precision. Such granular risk assessment forms the cornerstone for individualized treatment intensification or de-escalation, allowing clinicians to tailor therapeutic intensity based on molecular evidence rather than empirical protocols. This not only optimizes outcomes by ensuring that aggressive therapies are reserved for high-risk patients but also spares low-risk individuals from unnecessary toxicity, thereby enhancing quality of life.

However, the journey toward widespread clinical adoption is not without challenges. Technical standardization remains a significant hurdle, as variations in pre-analytical handling, assay platforms, and bioinformatic pipelines can impact reproducibility and comparability across studies. Clinically, robust validation through prospective, interventional trials is essential to establish ctDNA as a definitive biomarker for guiding treatment decisions. Moreover, the biological interpretation of ctDNA findings requires careful consideration—factors such as tumor heterogeneity, clonal evolution, and non-malignant sources of cell-free DNA can complicate the clinical utility of ctDNA results. Balancing these research perspectives necessitates a collaborative effort among laboratory scientists, clinical researchers, and regulatory bodies to develop consensus guidelines and evidence-based frameworks.

Looking ahead, the future of ctDNA in neoadjuvant therapy lies in addressing these challenges through multidimensional strategies. Pushing for technical standardization, such as harmonizing detection thresholds and establishing quality control metrics, will enhance reliability and facilitate broader implementation. Large-scale, interventional clinical studies are urgently needed to validate the predictive and prognostic value of ctDNA, particularly in guiding treatment modifications. Furthermore, integrating ctDNA with other multi-omics data—such as transcriptomic, proteomic, and immune profiling—will provide a more comprehensive view of tumor behavior and treatment response, enabling truly personalized therapeutic approaches.

Ultimately, the impact of ctDNA extends beyond neoadjuvant therapy, heralding a new era in cancer management characterized by real-time, dynamic, and precision-driven care. By enabling earlier detection of treatment failure or recurrence, ctDNA empowers clinicians to intervene proactively, potentially improving survival outcomes. Moreover, its role in de-escalating therapy for low-risk patients aligns with the growing emphasis on reducing treatment-related morbidity and preserving quality of life. As research continues to unravel the complexities of ctDNA biology and refine its clinical applications, this biomarker is poised to become a cornerstone of oncology practice, transforming the management of solid tumors from a one-size-fits-all approach to a tailored, patient-centric model. The convergence of technological advancements, rigorous clinical validation, and integrative data analysis will be pivotal in realizing the full potential of ctDNA, ultimately leading to better patient outcomes and a more efficient healthcare system. ctDNA provides a dynamic, phase-specific biomarker across the entire neoadjuvant continuum, enabling a shift from static evaluation to real-time, response-adapted precision oncology (**Table 3**).

**Table 3:** Clinical application framework of ctDNA across the neoadjuvant treatment continuum.

Clinical phase	ctDNA assessment timing	Detection purpose	Key readouts	Clinical interpretation	Decision-making impact	Evidence strength	Limitations	Recommended strategy
Baseline (Pre-treatment)	Before initiation of neoadjuvant therapy	Risk stratification + molecular profiling	- ctDNA positivity - ctDNA concentration (VAF, TF) - Mutation profile (EGFR, KRAS, PIK3CA, etc.)	- Positive ctDNA → high tumor burden, aggressive biology - High VAF → worse prognosis - Actionable mutations → therapy selection	- Select targeted therapy - Identify high-risk patients - Trial enrollment	High	- False negatives in low-shedding tumors - CHIP interference	Combine with imaging + tissue NGS; prefer tumor-agnostic panel initially
Early on-treatment	After 1–2 cycles (week 2–4)	Early response prediction	- ctDNA clearance - ≥50% VAF reduction	- Rapid clearance → strong responder - Persistent ctDNA → early resistance	- Early treatment adaptation - Switch regimen if non-response	High (breast, CRC, NSCLC)	- Assay sensitivity variability - Timing inconsistency	Serial sampling (baseline + week 3) recommended
Mid-treatment	Halfway through neoadjuvant therapy	Response monitoring + risk re-stratification	- ctDNA kinetics pattern (clearance/decline/persistence)	- Clearance → favorable trajectory - Slow decline → partial response - Persistent/rising → treatment failure	- De-escalation (responders) - Intensification (non-responders)	Moderate–High	- Lack of standardized thresholds	Integrate with imaging (RECIST/PET)
Preoperative (Post-NAT)	After completion of neoadjuvant therapy, before surgery	Predict pCR + detect MRD	- ctDNA clearance status - residual ctDNA level	- ctDNA-negative → high probability of pCR - ctDNA-positive → residual disease	- Surgical planning - Identify candidates for escalation	High	- Sensitivity still imperfect - Low shedding tumors	Combine with MRI/PET for best accuracy
Postoperative (MRD window)	~2–4 weeks after surgery	MRD detection	- ctDNA positivity (binary)	- Positive → occult residual disease - Negative → low recurrence risk	- Guide adjuvant therapy - Stratify recurrence risk	Very high	- False negatives possible - Timing critical	Prefer tumor-informed assay
Adjuvant therapy monitoring	During adjuvant therapy	Treatment efficacy evaluation	- ctDNA clearance or persistence	- Clearance → effective therapy - Persistent ctDNA → resistant disease	- Continue/switch therapy	Moderate	- Limited prospective evidence	Serial dynamic monitoring
Surveillance (follow-up)	Every 3–6 months	Early recurrence detection	- Re-emergence of ctDNA	- Molecular recurrence precedes imaging	- Early intervention window	Very high	- Optimal intervention unclear	Combine with imaging follow-up
At recurrence	At progression	Molecular profiling for salvage therapy	- New mutations - Clonal evolution	- Emergence of resistance mutations	- Guide targeted therapy - Avoid re-biopsy	High	- Tumor heterogeneity	Prefer broad NGS panel
